# Evolution of the repression mechanisms in circadian clocks

**DOI:** 10.1186/s13059-021-02571-0

**Published:** 2022-01-10

**Authors:** Jonathan Tyler, Yining Lu, Jay Dunlap, Daniel B. Forger

**Affiliations:** 1 Department of Mathematics, University of Michigan, Ann Arbor, 48109 MI USA; 2 Division of Pediatric Hematology/Oncology, Department of Pediatrics, University of Michigan, Ann Arbor, 48109 MI USA; 3 Department of Molecular and Systems Biology, Geisel School of Medicine at Dartmouth, Hanover, 03755 NH USA; 4 Department of Computational Medicine and Bioinformatics, University of Michigan, Ann Arbor, 48109 MI USA

**Keywords:** Circadian clocks, Evolution, Transcription, Protein sequestration, Phosphorylation

## Abstract

**Background:**

Circadian (daily) timekeeping is essential to the survival of many organisms. An integral part of all circadian timekeeping systems is negative feedback between an activator and repressor. However, the role of this feedback varies widely between lower and higher organisms.

**Results:**

Here, we study repression mechanisms in the cyanobacterial and eukaryotic clocks through mathematical modeling and systems analysis. We find a common mathematical model that describes the mechanism by which organisms generate rhythms; however, transcription’s role in this has diverged. In cyanobacteria, protein sequestration and phosphorylation generate and regulate rhythms while transcription regulation keeps proteins in proper stoichiometric balance. Based on recent experimental work, we propose a repressor phospholock mechanism that models the negative feedback through transcription in clocks of higher organisms. Interestingly, this model, when coupled with activator phosphorylation, allows for oscillations over a wide range of protein stoichiometries, thereby reconciling the negative feedback mechanism in *Neurospora* with that in mammals and cyanobacteria.

**Conclusions:**

Taken together, these results paint a picture of how circadian timekeeping may have evolved.

**Supplementary Information:**

The online version contains supplementary material available at (10.1186/s13059-021-02571-0).

## Background

Circadian clocks time most behavioral and physiological events in a 24-h day to optimize fitness in many organisms [1]. Molecular mechanisms are responsible for driving these rhythms at the cellular level [2]. A common theme for the generation of oscillations at the molecular level is the presence of negative feedback [3]. However, the mechanisms by which various organisms implement negative feedback differ. For example, in cyanobacteria, three proteins, KaiA, KaiB, and KaiC, interact to generate near 24-h rhythms in the phosphorylation status of KaiC, giving rise to a purely post-translational clock. On the other hand, eukaryotic organisms produce the required negative feedback through transcriptional activation of a repressor that, after a sufficient amount of repressor is present, inhibits the activator from further promoting transcription.

Despite our increased understanding of the core negative feedback architecture across many organisms, the evolutionary paradigm of circadian clocks remains unclear. Here, we develop novel mathematical models of the clocks in cyanobacteria, *Neurospora*, and mammals that reveal convergent principles by which diverse organisms generate oscillations. Our cyanobacterial clock models reflect protein sequestration between KaiA and KaiC [4, 5] while transcription regulates proper stoichiometric balance. Additionally, we present a mathematical model of the eukaryotic clock incorporating a novel “phospholock” mechanism that couples phosphorylation with protein sequestration. We find that the addition of phosphorylation increases the robustness of oscillations at biologically reasonable protein affinities. Furthermore, we show that, when adding activator phosphorylation, our model allows for oscillations at lower stoichiometric ratios of repressor to activator, reconciling experimental results from *Neurospora* with sequestration models.

Altogether, our analysis reveals that the circadian clock mechanisms among prokaryotes and eukaryotes may have evolved towards similar ends. That is, as the clock’s core feedback moved from post-translational regulation in cyanobacteria to a transcription-translation feedback loop in *Neurospora* and higher organisms, the regulation of stoichiometry, which is essential for robust oscillations, moved from a transcription-translation feedback loop to a post-translational mechanism.

## Results

### A detailed model of the cyanobacterial clock

**Fig. 1:**
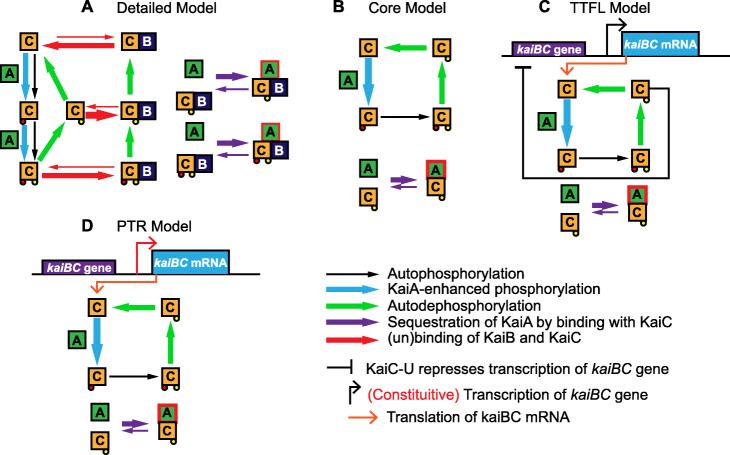
Reaction diagrams for the cyanobacterial clock models. Reaction diagrams for the detailed model (**A**), core model (**B**), TTFL model (**C**), and the PTR model (**D**). **A** Squares are proteins in various states; small circles indicate the phosphatase groups on the corresponding sites (red corresponds to the T432 site and yellow to the S431 site). Arrows indicate reactions among proteins, the width of which shows the relative strength. **B** The schematic of the core mechanism where KaiA enhances only the phosphorylation from *U* to *T*. **C** Extension of the core mechanism: the TTFL as an inhibition scheme where KaiC-S acts like an inhibitor of the *kaiBC* gene. **D** Schematic of the post-translational regulation (PTR) model with a constitutive source of transcription

Cyanobacteria are among the simplest organisms to exhibit circadian oscillations in cellular components. Proper functioning of the cyanobacterial circadian clock relies on interactions among three proteins: KaiA, KaiB, and KaiC [6]. Central to the protein interactions are two indispensable phosphorylation sites on KaiC-Ser-431 (S431) and KaiC-Thr-432 (T432). Recently, crystal structural analysis of the protein complexes in the KaiABC system revealed a detailed sequestration mechanism of KaiA by KaiBC protein complexes (Fig. 1A) and confirmed in silico [7, 8]. First, KaiA acts as an enzyme to enhance the phosphorylation of KaiC on the T432 site and, subsequently, the S431 site. Then, once site S431 is phosphorylated, KaiC undergoes a conformational change from a pre-hydrolysis state to a post-hydrolysis state creating a hub [9]. Simultaneously, KaiB undergoes a fold-change transition into an active state that is captured by the post-hydrolysis KaiC on the N-terminal (CI) domain. The KaiBC complex recruits KaiA proteins and prevents KaiC activation on the C-terminal (CII) domain. In other words, KaiBC complexes sequester free KaiA proteins.

Here, we model the sequestration mechanism of KaiA by the KaiBC complex with a system of ordinary differential equations (ODEs) assuming mass-action kinetics (see Eqs. –(8) in Additional file 1: Supplementary Information). Our model considers KaiC proteins in four states: unphosphorylated KaiC (denoted by *U*), S431 phosphorylated KaiC (denoted by *S*), T432 phosphorylated KaiC (denoted by *T*), and S431 and T432 phosphorylated KaiC (denoted by *ST*). A similar notation is used in the phosphorylation status of KaiBC. Our model assumes that KaiB binding only happens when the S431 site is phosphorylated [10, 11].

Simulations of this detailed model suggest that the components oscillate over a wide range of reaction rates. Analyzing the temporal profiles of the KaiABC oscillator, we see that the fraction of phosphorylated KaiC oscillates with a roughly 24-h period (Fig. S1, Additional file 1: Supplementary Information). Also, an asymmetry between the KaiC phosphorylation and dephosphorylation phases are consistent with previous experimental results [10, 12]. Analysis of the phases of these profiles suggests that the dynamics are indeed what we expect: first, free KaiA activates unphosphorylated KaiC (*U*); next, activated KaiC phosphorylates on the T432 site (*T*), which then slowly phosphorylates on the S431 site (*ST*). The total phosphorylation level of KaiC increases accordingly during the activation phase. Meanwhile, KaiB begins to bind with the S431 and T432 phosphorylated KaiC. The resulting KaiBC complexes in turn sequester KaiA through tight binding. Once sequestration depletes KaiA sufficiently, phosphorylation stops and dephosphorylation dominates, leading the system back to a highly unphosphorylated state.

**Fig. 2:**
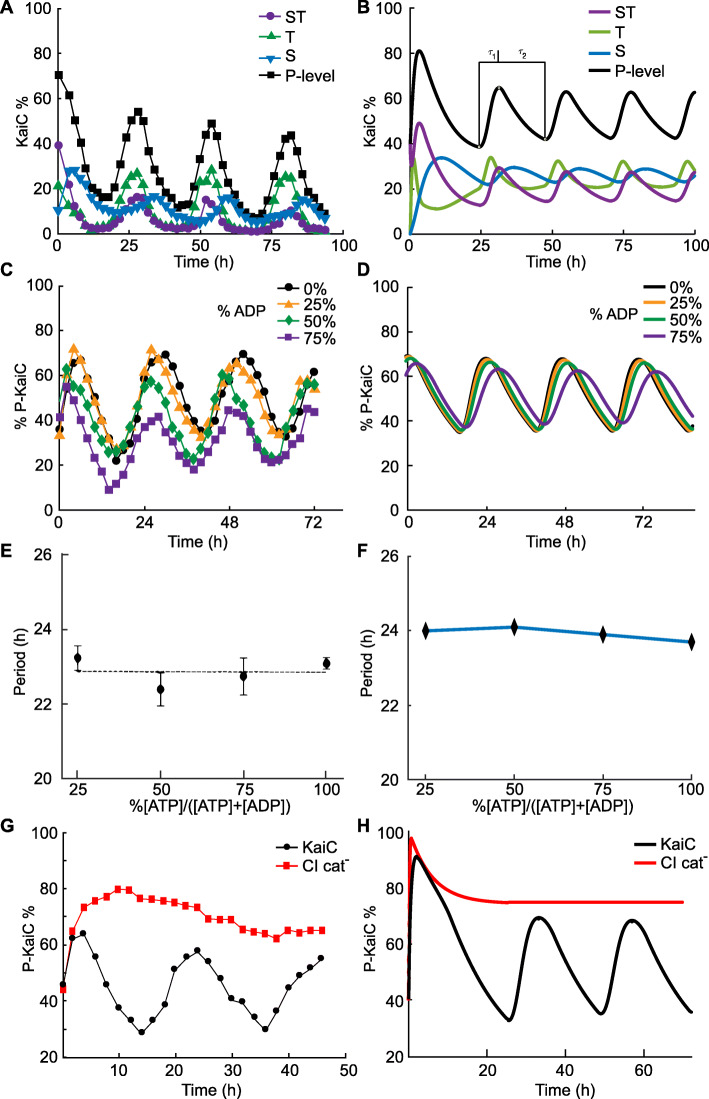
Simulations of the detailed model recapitulate experimental results. **A**, **B** Comparison with experimental data from Rust et al. [10]. In both results, *τ*_1_=9.5 h is the phosphorylation phase duration and *τ*_2_=18.5 h is the dephosphorylation phase duration. **C**–**F** Comparison with circadian data from Phong et al. [12], our model shows a robust period around 24 h under many ATP/ADP ratios. **G**, **H** Comparison with Phong et al. [12], where we confirmed the importance of the CI domain in sustaining oscillations. We simulate the model with weak KaiB-KaiC binding representing the KaiC muted in the CI domain (CI cat-) and the oscillation is abolished

Our detailed model reproduces important qualitative and quantitative results, which we illustrate by plotting circadian data from [12] alongside our model simulations (Fig. 2). In particular, we observe an asymmetric circadian rhythm where KaiC proteins spend less time in phosphorylation than dephosphorylation (Fig. 2A, B). Also, our model shows a robust period of around 24 h with less than 5% fluctuations for several ATP/ADP ratios (Fig. 2C–F). The CI domain of KaiC binds with KaiB, and thus sequesters KaiA, at night as shown experimentally in [12] (Fig. 2G–H). Taken together, our model recapitulates experimental results revealing the importance of the KaiC CI domain for the proper functioning of the cyanobacterial circadian clock.

Finally, we perform a bifurcation analysis (Fig. S2, Additional file 1: Supplementary Information) and stochastic simulations (Fig. S3, Additional file 1: Supplementary Information) of our detailed model. This bifurcation analysis reveals a result similar to that in [13] where the relative ratio of KaiA and KaiB to KaiC plays an important role in the circadian clock (Fig. S2, Additional file 1: Supplementary Information). Stochastic simulations reveal that, as the total number of KaiC molecules in the system increases, the stability of the oscillations increases. When the total number of KaiC molecules is low (*C*_*T*_≤50), the trajectories exhibit randomness without sustained oscillations (Fig. S3A, Additional file 1: Supplementary Information). As *C*_*T*_ increases, the stochastic simulations approach that of the deterministic system (Fig. S3, Additional file 1: Supplementary Information).

### A simple model of the cyanobacterial clock reveals tight binding is necessary for oscillations, similar to the mammalian system

Here, we propose a simple model of the cyanobacterial clock where only the KaiA and KaiC proteins are explicitly included (see Fig. 1B for the schematic). Our model is based on the detailed cyanobacterial model from above which accounts for a wide range of experimental observations. In our simplified model, given below in System (C), the KaiA-enhanced phosphorylation of the *T* site is much faster than autophosphorylation of the *S* site. The tight binding between KaiA and the unphosphorylated KaiC ensures that phosphorylation proceeds rapidly even in the presence of low amounts of free KaiA (the first equation in System (C)). As the phosphorylation continues, the number of KaiC proteins phosphorylated on the *S* site increases (the second equation in System (C)). In turn, sequestration of KaiA increases through tight-binding (the last equation in System (C)).

C $$ \begin{aligned} \frac{\mathrm{d}[T]}{\mathrm{d}t} &= k_{1}f([S], A_{T}, K_{d})(C_{T} - [T]-[ST]-[S]) - k_{2}[T] \\ \frac{\mathrm{d}[ST]}{\mathrm{d}t} &= k_{2}[T] - k_{3}[ST]\\ \frac{\mathrm{d}[S]}{\mathrm{d}t} &= k_{3}[ST]-k_{4}[S]\\ f([S], A_{T}, K_{d}) &= \left(A_{T} - [S] - K_{d} + \sqrt{(A_{T} - [S]-K_{d})^{2}+4K_{d}A_{T}}\right)/2 \end{aligned}  $$

The variable *C*_*T*_ is the total amount of the KaiC protein in the system. In the last equation, the free amount of KaiA, denoted by [*A*], is represented as a function of S431 phosphorylated KaiC that is derived under equilibrium assumptions similar to those in [14] (see Supplementary Information, Section 3 for a detailed derivation). As in the detailed model, the variable [*T*] gives the amount of KaiC protein phosphorylated on the *T* site. Similarly, the variables [*S*] and [*ST*] reflect the amount of KaiC protein phosphorylated on the *S* site and both the *S* and *T* sites, respectively.

**Fig. 3:**
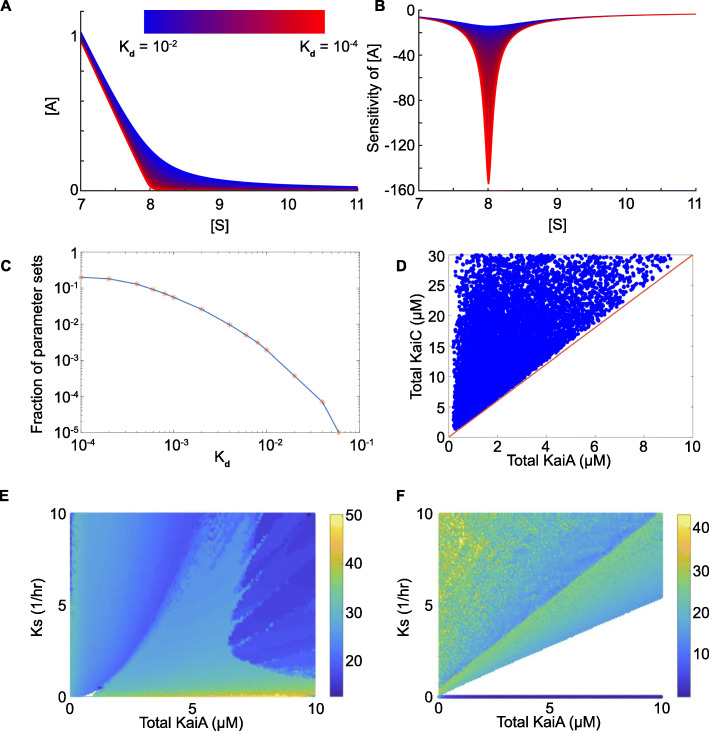
Simulations of core cyanobacterial models reveal keys to robust circadian oscillations. **A** Profile of the KaiA amount ([A]) with respect to the KaiC-S amount ([S]). As the curves move from blue to red, the binding affinity increases (i.e., *K*_*d*_ decreases from 10^−2^ to 10^−4^. Here, *A*_*T*_=8. **B** The sensitivity of the KaiA amount (**A**) with respect to the KaiC-S amount ([S]). Again, blue curves indicate weaker binding affinity while red curves reflect tighter binding. **C** The core model generates oscillations for a lower fraction of parameter sets as *K*_*d*_ increases from 10^−4^ to 10^−1^. The parameter sets are plotted as sample points (indicated by “*”) with a fitted curve on a log plot. **D** Any parameter set that generates oscillations is located above the line *C*_*T*_=1/3*A*_*T*_, verifying the balanced molar ratio condition. **E**, **F** Simulation of the TTFL model and the PTR oscillator model. Each oscillation is plotted as a point with a scaled color representing the period length. See Table S1 (Additional file 1: Supplementary Information) for a detailed description of the parameters

Our simulations demonstrate that KaiA sequestration through tight binding with S431 phosphorylated KaiC is indispensable for generating oscillations for various values of the binding affinity of KaiA and S431 phosphorylated KaiC (*K*_*d*_). In particular, as the *K*_*d*_ value decreases (i.e., the binding affinity increases), the cusp of the KaiA concentration sharpens, and the magnitude of the sensitivity of the KaiA concentration to the amount of *S* increases (Fig. 3A, B). Increasing sensitivity, or order of reaction [15], of the activator KaiA with respect to S431 phosphorylated KaiC corresponds to an increasing likelihood of oscillations [15, 16]. Moreover, for increasing values of *K*_*d*_, the fraction of parameter sets that generate oscillations decreases (Fig. 3C). This result shows that the system is more likely to generate stable oscillations when there is stronger KaiA sequestration (i.e., smaller *K*_*d*_). A similar dependence on smaller *K*_*d*_ values is present in the interaction of the activator and the repressor in the mammalian system [14].

### A necessary stoichiometric ratio for oscillations in the simple cyanobacterial model

Additional theoretical analysis (see Supplementary Information, Section 3) and simulations confirm that a necessary condition for oscillations to occur in our model is

$$ C_{T} > (1+r)A_{T} + \epsilon, $$

where *r* is a constant related to the rates of phosphorylation and dephosphorylation, and *ε* is a small positive constant. Consistent with existing experimental and modeling results [13, 17, 18], our simulations suggest that a balanced molar ratio between KaiA and KaiC abundance is crucial for generating sustainable oscillations. The observation that KaiB abundance does not affect the circadian rhythm in the same way is also consistent with the model design. In our simulations, we choose parameter values such that *r*=2 and *ε*<<1. The condition effectively turns into the linear criterion:

1 $$ C_{T} > 3A_{T}.  $$

Simulation results verify that parameters violating Condition (1) rarely generate oscillations (Fig. 3D). A similar mechanism has been proposed for the mammalian circadian clock where repressors and activators are in a balanced molar ratio [14].

### Simple cyanobacterial model is functionally equivalent to the previous Kim-Forger model

System (C) reflects the interplay between phosphorylation and sequestration in the KaiABC oscillator in cyanobacteria. Correctly timed and ordered phosphorylation is necessary for robust circadian rhythm generation. Surprisingly, even though the mechanisms that generate rhythms are different among cyanobacteria and higher-order eukaryotes, the form of the repression function is similar. In particular, recall the simple Kim-Forger model of the mammalian circadian clock from [14, 19]:

M $$ \begin{aligned} \frac{\mathrm{d}M}{\mathrm{d}t} &= \alpha_{1}f(P,A,K_{d}) - \beta_{1}M \\ \frac{\mathrm{d}P_{c}}{\mathrm{d}t} &= \alpha_{2}M - \beta_{2}P_{c}\\ \frac{\mathrm{d}P}{\mathrm{d}t} &= \alpha_{3}P_{c}-\beta_{3}P\\ f(P,A,K_{d}) &= \left(A - P - K_{d} + \sqrt{(A -P-K_{d})^{2}+4{AK}_{d}}\right)/(2A) \end{aligned}  $$

Here, *M* models the mRNA of the repressor protein, *P*_*C*_ is the cytosolic repressor protein, and *P* is the nuclear repressor protein. The parameter *K*_*d*_ is the binding affinity of the activator *A* and the nuclear repressor, *P*. The function *f*(*P*,*A*,*K*_*d*_) models the amount of free activator as a function of total activator, estimated by *A*, and repressor (*P*) present [14]. Note that the repression functions in Systems (C) and (M) are equivalent, indicating that the action of repression is conserved across the organisms even though the specific mechanisms of repression are different.

### Additional transcription-translation feedback loop adds robustness to oscillations in cyanobacteria

We also find that an additional transcription-translation feedback loop (TTFL) plays a significant role in sustaining robust oscillations. Besides the KaiABC system, the histidine kinases SasA and CikA regulate the output signaling from the post-translational oscillator to the transcriptional activity in the in vivo oscillator. The integrated roles of SasA, CikA, and RpaA together with KaiABC in the cyanobacterial circadian clock were summarized recently [20]. When KaiC phosphorylation reaches its peak, SasA binds to the CI domain of the ST-phosphorylated KaiC [9, 21], autophosphorylates, and transfers the phosphate group to RpaA, turning on the transcription factor [22]. During the dephosphorylation phase, when KaiB binds to the CI domain to form the KaiBC complex, SasA is released from KaiC. CikA is then recruited by the KaiBC complex, dephosphorylates RpaA, and thus, inhibits the transcription. In other words, the transcription of the *kaiBC* gene is activated when most of the KaiC proteins are highly phosphorylated and is inhibited when KaiC proteins are mostly in the *S* state. Therefore, we model the TTFL as an inhibition scheme where S431 phosphorylated KaiC acts like an inhibitor of the *kaiBC* gene (Fig. 1C, System (TTFL) below).

TTFL $$ \begin{aligned} \frac{\mathrm{d}[M]}{\mathrm{d}t} &= V_{trsp}\frac{100}{1+\left(\frac{[S]}{K_{0}}\right)^{4}} - V_{m}[M] \\ \frac{\mathrm{d}[U]}{\mathrm{d}t} &= K_{s}[M] -k_{1}[A][U] + k_{4}[S]-V_{d}[U]\\ \frac{\mathrm{d}[T]}{\mathrm{d}t} &= k_{1}[A][U] - k_{2}[T]-V_{d}[T]\\ \frac{\mathrm{d}[ST]}{\mathrm{d}t} &= k_{2}[T]-k_{3}[ST]-V_{d}[ST]\\ \frac{\mathrm{d}[S]}{\mathrm{d}t} &= k_{3}[ST]-k_{4}[S]-V_{d}[S] \\ [A] &= \left (A_{T} - [S] - K_{d} + \sqrt{(A_{T}-[S]-K_{d})^{2}+4K_{d}A_{T}}\right)/2 \qquad \text{TTFL}\end{aligned}  $$

The variable *M* is the amount of mRNA transcribed from the *kaiBC* gene.

We investigate the role of the transcription-translation feedback loop (System (TTFL)) as an additional negative feedback loop in the KaiC system by comparing with a modified system with a constitutive source of transcription (Fig. 1D, System (PTR) below). See Table S1 (Additional file 1: Supplementary Information) for a detailed description of the parameters and the ranges of parameters used.

PTR $$ \begin{aligned} \frac{\mathrm{d}[M]}{\mathrm{d}t} &= V_{trsp} - V_{m}[M] \\ \frac{\mathrm{d}[U]}{\mathrm{d}t} &= K_{s}[M] -k_{1}[A][U] + k_{4}[S]-V_{d}[U]\\ \frac{\mathrm{d}[T]}{\mathrm{d}t} &= k_{1}[A][U] - k_{2}[T]-V_{d}[T]\\ \frac{\mathrm{d}[ST]}{\mathrm{d}t} &= k_{2}[T]-k_{3}[ST]-V_{d}[ST]\\ \frac{\mathrm{d}[S]}{\mathrm{d}t} &= k_{3}[ST]-k_{4}[S]-V_{d}[S] \\ [A] &= \left (A_{T} - [S] - K_{d} + \sqrt{(A_{T}-[S]-K_{d})^{2}+4K_{d}A_{T}}\right)/2 \qquad \text{PTR} \end{aligned}  $$

We simulate both Systems (TTFL) and (PTR) with 22,500 randomly generated parameter sets varying the transcription rate, *K*_*s*_, and the total KaiA concentration, *A*_*T*_. The TTFL model generates circadian oscillations under a wide range of parameters (98.84%, Fig. 3E), while the PTR model, without the additional feedback loop, only exhibits oscillations when satisfying Condition (1) (60.33%, Fig. 3F). Altogether, our analysis and simulations suggest that the TTFL, as an additional negative feedback loop in the cyanobacterial clock, can help sustain the required molar ratio balance through a homeostatic mechanism.

### A novel “Phospholock” model of the eukaryotic clock

In light of recent results that reveal a complex interplay between binding of the activator and repressor as well as the timing and ordering of phosphorylation of the repressors [23–25], we introduce an extension of System (M) for higher-order eukaryotes that incorporates additional phosphorylation of the repressor after binding to the activator. In particular, the repressor complex, after binding with the activator, is phosphorylated, subsequently leading to differential binding affinities between the activator and repressor complexes at various phosphorylation states and dissociation of the two complexes occurring after sufficient phosphorylation. We call this mechanism the “phospholock” as the additional phosphorylation of the repressor in turn keeps the activator and repressor complex sequestered together more so than pure protein sequestration. Notably, after phosphorylation is incorporated, the mathematical model of the repression mechanism is equivalent to those Systems (C) and (M) up to additional coefficients as shown by the model below (System (E)).

In this phospholock model, the activator complex, A, promotes the transcription of the repressor gene. The repressor mRNA, M, is then translated to the repressor protein r, which forms a final complex R with other proteins, e.g., CK1. Next, the repressor complex binds to A, leading to the subsequent phosphorylation of the repressor complex. After sufficient phosphorylation, the activator and repressor complexes dissociate, leaving the activator free to promote transcription of the repressor gene again. In this way, we incorporate phosphorylation, which has been shown to be essential to oscillations, with the previously established protein sequestration repression mechanism.

We describe the phospholock mechanism with a simple model below (see Supplementary Information, Section 4, for the derivation of the repression function, *f*(*R*)).

E $$ \begin{aligned} \frac{\mathrm{d}\mathrm{M}}{\mathrm{d}t} &= \alpha_{1} f(\mathrm{R}) - \beta_{1} \mathrm{M}\\ \frac{\mathrm{d}\mathrm{r}}{\mathrm{d}t} &= \alpha_{2} \mathrm{M} - \beta_{2} \mathrm{r} \\ \frac{\mathrm{d}\mathrm{R}}{\mathrm{d}t} &= \alpha_{3} \mathrm{R} - \beta_{3} \mathrm{R} \\ f(\mathrm{R}) &= \frac{\tilde{K}_{1} \cdot A_{T}-{\tilde{K}_{1}}\cdot \mathrm{R}-{\tilde{K}_{2}} + \sqrt{({\tilde{K}_{1}} \cdot A_{T}+{\tilde{K}_{1}}\cdot \mathrm{R}+{\tilde{K}_{2}})^{2}-4{\tilde{K}_{1}}^{2}A_{T}\mathrm{R}}}{2{\tilde{K}_{1}}}. \end{aligned}  $$

**Fig. 4:**
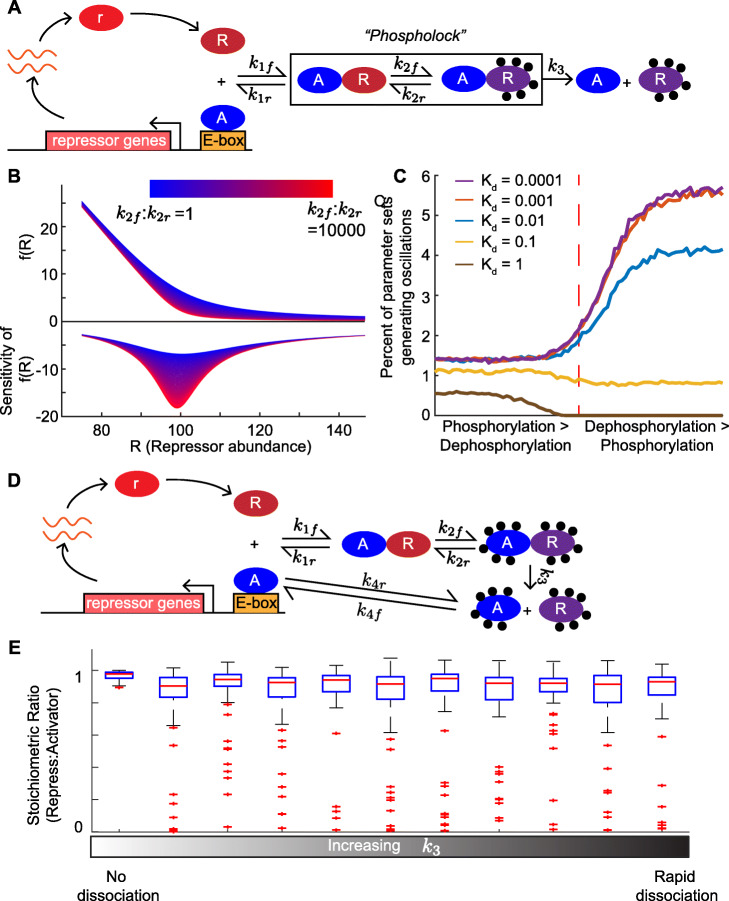
Novel “phospholock” model of the eukaryotic circadian clock. **A** Schematic of the “phospholock” model of the eukaryotic circadian clock. The activator complex, A, promotes transcription of the repressor gene, which is then translated to the repressor protein r. Next, the repressor protein(s) form complexes with other proteins, specifically kinases. Then, the repressor complex binds to the activator complex. Over time, the repressor proteins become gradually more phosphorylated until it dissociates, leaving the activator complex free, and thus completing the cycle. **B** The repression function *f*(*R*) (top) from System (E) and the sensitivity (bottom) for a specific parameter set (see the “Methods” section) with *K*_*d*_=1. The ratio of phosphorylation to dephosphorylation is increased from 1 (blue) to 10000 (red). As the ratio increases, the cusp of the repression function sharpens, reflected in the increase in the magnitude of the peak sensitivity (bottom). **C** The percent of parameter sets that exhibit oscillations increases with increasing phosphorylation strength and varying *K*_*d*_ values (0.0001, 0.001, 0.01, 0.1, and 1). When the *K*_*d*_ values are small (≤ 0.01), the system is more likely to generate oscillations when dephosphorylation is stronger than phosphorylation. However, at more likely *K*_*d*_ levels, i.e., *K*_*d*_≥0.1, the system is more likely to generate oscillations when phosphorylation is stronger than dephosphorylation. **D** Schematic of the phospholock mechanism with the additional phosphorylation of the activator (as in the *Neurospora* system). **E** The distribution of stoichiometric ratios (R:A) calculated from parameter sets that generate oscillations for increasing *k*_3_ values. Adding phosphorylation of the activator increases the range of stoichiometric ratios from parameter sets that exhibit oscillations as the *k*_3_ parameter increases

In Fig. 4B, we plot the repression function *f*(*R*) for a specific parameter set where *K*_*d*_=1 while varying the ratio of phosphorylation to dephosphorylation (i.e., $k_{2} = {\frac {k_{2f}}{k_{2r}}}$). As *k*_2_ increases, the cusp of the repression function sharpens (Fig. 4B, top). Moreover, the magnitude of the peak sensitivity increases as *k*_2_ increases (Fig. 4B, bottom).

Next, we investigate how the interplay between the *K*_*d*_ value and the ratio of the strength of phosphorylation to dephosphorylation (*k*_2_) affects the robustness of oscillations. As *K*_*d*_ values decrease, the likelihood that the system generates oscillations increases in general (Fig. 4C). Moreover, at small *K*_*d*_ values, the system is more likely to generate oscillations when dephosphorylation is stronger than phosphorylation, i.e., *k*_2_<1. However, as *K*_*d*_ increases, e.g., *K*_*d*_≥0.1, the system is more likely to generate oscillations when phosphorylation is stronger than dephosphorylation, i.e., *k*_2_>1 (Fig. 4C).

### Additional activator phosphorylation in the phospholock model allows for wider stoichiometric ratios

While System (E) represents the mechanisms of the circadian clock in Drosophila and mammals, we now investigate the mechanism in *Neurospora*. The repression mechanism in the *Neurospora* circadian clock historically has been regarded as a Hill-type repression mechanism over a pure protein sequestration mechanism [19]. In this Hill-type repression, the repressor inactivates the activator by catalyzing several successive phosphorylations [26]. Moreover, the higher number of phosphorylations results in a higher chance of oscillations occurring [19]. Even though the assumptions used to derive the Hill function are very restrictive [16], mathematical models of the *Neurospora* clock [27–29] continue to use a Hill-type repression term to model the mechanism by which FRQ represses WCC over protein sequestration for two key reasons: 1) phosphorylation of the activator is a crucial element in the generation of oscillations [30–32], and 2) the stoichiometric ratio of repressor to activator in *Neurospora* is not consistent with required stoichiometric ratios in protein sequestration models [33].

However, when we add phosphorylation of the activator to the phospholock model (Fig. 4D), we find that oscillations are possible at a wide range of stoichiometric ratios. System (E), now with added phosphorylation of the activator, becomes

N $$ {}\begin{aligned} \frac{\mathrm{d}\mathrm{M}}{\mathrm{d}t} &= \alpha_{1} f(\mathrm{R}) - \beta_{1} \mathrm{M}\\ \frac{\mathrm{d}\mathrm{r}}{\mathrm{d}t} &= \alpha_{2} \mathrm{M} - \beta_{2} \mathrm{r} \\ \frac{\mathrm{d}\mathrm{R}}{\mathrm{d}t} &= \alpha_{3} \mathrm{R} - \beta_{3} \mathrm{R} \\ f(\mathrm{R}) &= \frac{\tilde{K}_{1}A_{T}-\tilde{K}_{3}R-\tilde{K}_{2}(1+k_{4}) + \sqrt{(\tilde{K}_{1}A_{T}+\tilde{K}_{3}R+\tilde{K}_{2}(1+k_{4}))^{2}-4\tilde{K}_{1}\tilde{K}_{3}A_{T}R}}{2\tilde{K}_{1}(1+k_{4})}. \end{aligned}  $$

Note that, even with the additional phosphorylation of the activator, the repression functions in Systems (C), (E), and (N) are equivalent up to changes in parameter coefficients. See Supplementary Information, Section 5, for the derivation of the repression function.

Next, we simulate thousands of parameter sets that generate oscillations for increasing *k*_3_ values and then compute the stoichiometric ratio given by each parameter set. As *k*_3_ increases above zero, the range of stoichiometric ratios that sustain oscillations increases (Fig. 4E). In fact, the stoichiometric ratio can attain values very close to zero (Fig. 4E), values well within the range of measured levels of nuclear FRQ and WCC in *Neurospora* [34]. Thus, adding in the phosphorylation of the activator can significantly decrease the number of repressors required for the system to exhibit oscillations. In this case, phosphorylation acts to inactivate WCC without requiring the presence of FRQ. Moreover, increasing *k*_3_ actually decreases the robustness of the oscillations in this case (see Supplementary Information, Section 6).

## Discussion

Here, we present mathematical models of the cyanobacterial, mammalian, and Neurospora circadian clocks that reflect the crucial interplay between phosphorylation and sequestration of activators and repressors. Our models ultimately reveal a common repression function that generates oscillations at the molecular level independently in the three systems. This consistent repression function is particularly surprising considering the divergent mechanisms by which oscillations are generated, e.g., purely post-translational (cyanobacteria) versus a transcription-translation feedback loop (eukaryotes).

Moreover, our results reveal stoichiometric balancing requirements for oscillations and how these requirements may be satisfied in the three systems. For example, an additional transcription-translation feedback loop (TTFL) in cyanobacteria plays a significant role in sustaining robust oscillations by maintaining the required molar ratio (Condition (1)). This additional feedback loop matches the proposed role of the Rev-erb *α* TTFL in the mammalian clock [14, 19] that acts synergistically with the core feedback loop to maintain the molar ratio between activators and repressors. For example, when the molar ratio is perturbed to greater than 1 (i.e., more repressor), the additional feedback loop promotes activator production, restoring the 1:1 molar ratio required for robust oscillations [19]. Similarly, when the molar ratio is perturbed in the cyanobacterial system to greater than its required range (i.e., more free KaiC), then RpaA is dephosphorylated leading to low transcription of the *kaiBC* genes and a decrease in free KaiC, restoring the molar ratio required for robust oscillations.

To compare oscillations across species, we do not specifically account for the structure of some clock complexes. Accounting for this in molar ratios (e.g., the KaiC hexamer) shows that the requirements we see are consistent with other previous work (e.g., [18]). That being said, the mechanisms we discuss are far from a complete picture of circadian timekeeping. In fact, factors like co-operative binding or ultrasensitivity also play key roles [35, 36].

In *Neurospora*, it has been shown experimentally that phosphorylation of the activator is crucial to the generation of rhythms, and stoichiometric ratios of the repressor to activator do not agree with the ranges required from the protein sequestration paradigm. However, when additional phosphorylation of the activator is added to the phospholock model, the reconciliation among the possible models emerges. Additional phosphorylation of the activator allows for another method by which the repressor may inactivate the activator, namely successive phosphorylations. Once the activator is phosphorylated, it must be dephosphorylated to promote transcription again. Thus, fewer repressors are required to attain the required amount of inactivation of the activator than when there is no phosphorylation of the activator. That is, with no phosphorylation, once the repressor is degraded, free activators may promote transcription, so another repressor is required to sequester the activator. However, if the activator is phosphorylated, then another repressor does not need to bind to the activator. Thus, fewer repressors are required leading to a wider range in the stoichiometric ratios that generate oscillations.

Recent work using mice showed biochemically that PER promotes the nuclear entry of CRY and the phosphorylation of CLOCK [37]. This additional phosphorylation of CLOCK prevents it from binding to DNA, resulting in what the authors call “displacement-type repression” [37]. This is mechanistically identical to previous work showing that FRQ-mediated phosphorylation of specific sites on WC-1 and WC-2 is required for repression of WCC [32]. The two mechanisms may be identical, i.e., phosphorylation of the activator complex effected by the repressor complex brings about repression of the activator by preventing it from binding to DNA.

In previous protein sequestration models, the robustness of oscillations relied heavily on a tight binding affinity between the activator and repressor. In this way, the repressor sufficiently sequesters the activator, rendering it unable to promote transcription. However, these previous models required unrealistic binding affinities. In the phospholock model, the inactivation of the activator by the repressor is balanced between binding of the two components and phosphorylation/dephosphorylation of the repressor. That is, at low binding affinities of the activator and repressor, the phospholock model reveals that stronger phosphorylation adds robustness to the oscillations by keeping the activator inactivated. In contrast, at high binding affinities, stronger dephosphorylation adds robustness by freeing some activators to promote transcription.

The phospholock model also revealed that rapid degradation of the repressor is not required for sustained oscillations, confirming previous experimental results [23, 24, 38]. That is, simulations revealed that decreasing the *k*_3_ value, a surrogate marker of the degradation strength of the phosphorylated repressor, increases the sensitivity of the repression function, thereby increasing the robustness of oscillations. Therefore, our results are consistent with the important feature, now shown experimentally in both *Neurospora* [38] and mice [23], that rapid degradation of the phosphorylated repressors is not required for rhythm generation.

Altogether, our novel models of the cyanobacteria and eukaryotic circadian clocks corroborate the emerging theory that molecular clock mechanisms in divergent organisms may have evolved convergently [39]. In particular, a simple mechanism of the cyanobacteria clock is as follows: KaiA is required for the phosphorylation of KaiC on the *T* site. When the subsequent *S* site of KaiC is phosphorylated, KaiC binds to KaiB and gets “activated”. The “activated” KaiC can then bind and inactivate KaiA with the help of KaiB. We assume that KaiC, when bound to KaiB, is very efficient in inhibiting KaiA, such that effectively all KaiA is sequestered even in the presence of a small amount of KaiC with the *S* site phosphorylated. After the phosphorylation is completed and KaiC gets dephosphorylated at the *S* site, there is eventually not enough KaiC to sequester all KaiA, and KaiA is released. The free KaiA then efficiently phosphorylates the unphosphorylated KaiC, starting the cycle again. The eukaryotic systems work via a similar mechanism. An activator complex, A, activates the transcription of repressor proteins, which is functionally identical to KaiA activating KaiC on the *T* site. Next, the eukaryotic repressor proteins sequester and inactivate the activating complex. Similarly, the phosphorylation of KaiC on the *S* site allows KaiC to sequester and inactivate KaiA. Finally, the repressor proteins in eukaryotes undergo several phosphorylations and then dissociate, freeing the activating complex, completing the cycle. Similarly, as KaiC is dephosphorylated below a certain threshold, it releases KaiA, which then facilitates KaiC phosphorylation on the *T* site, completing the cycle. Despite differences among the three clocks, it is surprising that the systems’ core mechanisms share a similar architecture for generating robust oscillations.

Follow-up work could consider simulated evolution in silico experiments that may reveal efficient and robust mechanisms for generating oscillations across independent species. Such mechanisms could help further delineate how clocks may have evolved. Additionally, the plant molecular circadian clock is much more complex than that found in other organisms, at least in its TTFL structure. Further work should explore how sequestration and the mechanisms to balance molar ratios we discuss may play a role in the plant circadian clock. Further experiments could measure or change the molar ratios of activators and repressors to determine how the phospholock mechanism we propose extends the range where sustained oscillations are found.

## Conclusion

Our mathematical models reveal circadian clocks in cyanobacteria and some eukaryotes employ both protein sequestration and phosphorylation to generate oscillations. An additional transcription-translation feedback loop in cyanobacteria adds robustness to the oscillations over constitutive transcription of the *kaibc* genes. Interestingly, the Vrille or Rev-erb transcription-translation feedback loops in Drosophila and mammals, respectively, may have rediscovered this cyanobacterial mechanism to add robustness oscillations. In fungi and mammals, phosphorylation acts as a lock to keep repressor and activator complexes coupled and thus adds robustness to the oscillations, especially at higher *K*_*d*_ values (lower protein affinity). Furthermore, the phospholock in *Neurospora* includes phosphorylation of the activator, which drives wider stoichiometric ratios of repressor to activator. Thus, our model reveals how *Neurospora* fits into the previous protein sequestration model for mammalian circadian clocks. Taken together, these results paint a picture of how circadian timekeeping may have evolved [40–42].

## Methods

### Computing the fraction of parameter sets for which System (C) generates oscillations

For 14 *K*_*d*_ values increasing from 10^−4^ to 10^−1^, we randomly generate 10^5^ parameter sets based on the parameter values in Table S1 (Additional file 1: Supplementary Information). In particular, we fix the *k*_2_, *k*_3_, *k*_4_, *V*_*trsp*_, *V*_*d*_, and *V*_*m*_ parameters and select all other parameters from a random uniform distribution based on the ranges in Table S1 (Additional file 1: Supplementary Information). Then, we use the fast Fourier transform (fft) function in MATLAB to assess whether the parameter sets are sufficient for System (C) to exhibit oscillations.

Next, we simulate the TTFL and PTR models each 22,500 times after randomly generating parameter sets when varying *K*_*s*_ and *A*_*T*_ concentrations based on the ranges found in Table S1 (Additional file 1: Supplementary Information). We calculate the period by first applying the FFT on the time course and then identifying the strongest frequency in the spectrum.

### Plotting sensitivity of repression functions

We plot the repression function *f*([*S*],*A*_*T*_,*K*_*d*_) with respect to [*S*] from System (C) for decreasing values of *K*_*d*_ (10^−2^ to 10^−4^) using the parameter values in Table S1 (Additional file 1: Supplementary Information) (Fig. 3A). Additionally, in Fig.3B, we plot the sensitivity of *f*([*S*],*A*_*T*_,*K*_*d*_) (equal to ${\frac {\mathrm {d}\log f([S], A_{T}, K_{d})}{\mathrm {d}\log [A]}}$ [15, 16]) computed in MATLAB R2020b.

Similarly, we plot the repression function *f*(*R*) from System (E) using the parameter values found in Table 1 for $k_{2} = \frac {k_{2f}}{k_{2r}}$ values ranging from 1 to 10^4^ (Fig. 4B, top) for repressor amounts from 75 to 150. We also plot the sensitivity of *f*(*R*) (equal to ${\frac {\mathrm {d}\log f(R)}{\mathrm {d}\log R}}$ [15, 16]) for the same range of repressor values and *k*_2_ values (Fig. 4B, bottom) computed in MATLAB R2020b.

**Table 1:** Parameter values used to plot the repression function *f*(*R*) and its sensitivity with respect to *R* (Fig. 4B)

Parameter	Value
*α*_1_	95.2
*β*_1_	25.6
*α*_2_	43.4
*β*_2_	28.7
*α*_3_	98.3
*β*_3_	20.1
*K*_*d*_	1
*k*_3_	1.9
*A*_*T*_	98.9

### Counting parameter sets for which the phospholock model generates oscillations

We randomly generated 10^4^ parameter sets where *A*_*T*_, *α*_1_, *β*_1_, …, *α*_3_, and *β*_3_ were sampled from a uniform distribution between 0 and 100. Then, we sampled the parameters *k*_1*f*_ and *k*_2*f*_ from a uniform distribution between 0 and 1. To guarantee *K*_*d*_=1, we set *k*_1*r*_=*k*_1*f*_. Additionally, we sampled *k*_3_ from a uniform distribution between 0 and 0.1.

Next, we simulated the phospholock model for each randomly generated parameter set using the function ode23tb in MATLAB. Specifically, we randomly initialized the initial conditions and ran the model for 300 time units. Then, we took the mean of the M values and simulated the model again. The second time, however, we ran an event location procedure where the ode solver saves all times that satisfy: (1) *M* is equal to the mean calculated from the initial run and (2) *M* is increasing. Then, we concluded that the model oscillated if

$$ |({te}_{1}-{te}_{2}) - ({te}_{2} - {te}_{3})| < 0.001, $$

where *te*_1_ is the last time that the event took place, *te*_2_ is the second to last, and *te*_3_ is the third to last. We checked several plots of the oscillating parameter sets to make sure that our procedure selected parameter sets that generate oscillations.

Finally, we simulated the 10^4^ randomly generated parameter sets varying the phosphorylation strengths by setting *k*_2*r*_=100·*k*_2*f*_ (strongest dephosphorylation, Fig. 2C; left), *k*_2*r*_=10·*k*_2*f*_, *k*_2*r*_=*k*_2*f*_, …, *k*_2*r*_=0.0001·*k*_2*f*_ (strongest phosphorylation, Fig. 2C; right). Each time, we counted how many parameter sets generated oscillations.

### Stoichiometric ratio analysis

Beginning with *k*_3_=0, we randomly generate parameter sets until we recovered 100 such that the phospholock model with added activator phosphorylation generates oscillations (see Eqn. (56) in Supplementary Information for the updated repression function in this case). In particular, we randomly sample *A*_*T*_, *α*_1_, *β*_1_, …, *α*_3_, and *β*_3_ from a uniform distribution between 0 and 100. Next, we sample *k*_1*f*_, *k*_2*r*_, and *k*_4*f*_ from a uniform distribution between 0 and 1. We set *K*_*d*_=10^−5^ in this case, so *k*_1*r*_=10^−5^·*k*_1*f*_. Additionally, we assume phosphorylation is stronger than dephosphorylation in the phospholock, so we set *k*_2*f*_=10·*k*_2*r*_. Finally, we set autophosphorylation and autodephosphorylation as equal, so *k*_4*r*_=*k*_4*f*_. Parameter sets were assessed for whether they generated oscillations based on the same method as above.

For each of the 100 parameter sets that exhibit oscillations, we compute the stoichiometric ratio in the following way. First, we solve the following equation for R.

2 $$ \frac{A_{T} - \tilde{K}_{3}\cdot AR}{1+k_{4}} = \frac{\beta_{1} \beta_{2} \beta_{3} R}{ \alpha_{1} \alpha_{2} \alpha_{3}},  $$

where

$$ {}AR = \frac{\tilde{K}_{1}A_{T}+\tilde{K}_{3}R_{T}+\tilde{K}_{2}(1+k_{4}) - \sqrt{(\tilde{K}_{1}A_{T}+\tilde{K}_{3}R_{T}+\tilde{K}_{2}(1+k_{4}))^{2}-4\tilde{K}_{1}\tilde{K}_{3}A_{T}R_{T}}}{2\tilde{K}_{1}\tilde{K}_{3}}. $$

The solution to Eqn. (2) gives the repressor concentration at the steady state. Then, the stoichiometric ratio is

$$ S = \frac{R}{A_{T}}, $$

where R solves Eqn. (2).

## Supplementary Information

**Additional file 1** A separate pdf file that includes more detailed mathematical derivations and analysis of the models presented in the main text, Figures S1-S4, and Tables S1 and S2.

**Additional file 2** Review history.

## Data Availability

The codes for the phospholock analyses are provided in the GitHub repository (https://github.com/jptyler/Phospholock) under MIT license [43].
