# Supplementary material for: Evolution of the repression mechanisms in circadian clocks
Source: Genome Biol. 2022 Jan 10;23:17. doi: 10.1186/s13059-021-02571-0 (PMC8751359; doi:10.1186/s13059-021-02571-0)
Supplement: Supplementary file 1 — Additional file 1 A separate pdf file that includes more detailed mathematical derivations and analysis of the models presented in the main text, Figures S1-S4, and Tables S1 and S2. [file 13059_2021_2571_MOESM1_ESM.pdf]

Supplementary Information for "Evolution of the transcriptional mechanisms in circadian clocks"

Jonathan Tyler, Yining Lu, Jay Dunlap, Daniel Forger

October 2020

Contents

1 Description of the detailed Cyanobacterial model **1**

2 Bifurcation analysis and stochastic simulations **4**

3 Linear stability analysis of the core model **4**

4 Derivation of the repression function in the novel phospholock model **10**

5 Derivation of the repression function in the adjusted phospholock model with activator phosphorylation **12**

6 Sensitivity of transcription-rate function decreases with increasing k_3 values **14**

1 Description of the detailed Cyanobacterial model

To describe the full model, we list all of the reactions in Network (S1). Consistent with the main text, we consider KaiC proteins in four states: unphosphorylated KaiC (denoted by U), S431 phosphorylated KaiC (denoted by S), T432 phosphorylated KaiC (denoted by T), and double phosphorylated KaiC (denoted by ST). The notation for the KaiBC complexes are then $B \cdot U$, $B \cdot S$, $B \cdot T$, and $B \cdot ST$. See Table S1 for a detailed description of the parameters.

The reactions in the detailed model are:

$$
\begin{gathered}
\mathrm{U}+\mathrm{A} \xrightarrow{\mathrm{k}_1^f} \mathrm{AU}, \quad \mathrm{U} \xleftarrow{\mathrm{k}_1^b} \mathrm{AU} \xrightarrow{\mathrm{k_{cat}}} \mathrm{T} \\
\mathrm{T}+\mathrm{A} \xrightarrow{\mathrm{k}_2^f} \mathrm{AT}, \quad \mathrm{T} \xleftarrow{\mathrm{k}_2^b} \mathrm{AT} \xrightarrow{\mathrm{k_{cat}}} \mathrm{ST} \\
\mathrm{U} \xrightarrow{\mathrm{k_{ps}}} \mathrm{T} \xrightarrow{\mathrm{k_{ps}}} \mathrm{ST} \\
\mathrm{U}+\mathrm{B} \underset{\mathrm{k_1^{off}}}{\overset{\mathrm{k_1^{on}}}{\rightleftharpoons}} \mathrm{B}\cdot\mathrm{U}, \quad \mathrm{S}+\mathrm{B} \underset{\mathrm{k_2^{off}}}{\overset{\mathrm{k_2^{on}}}{\rightleftharpoons}} \mathrm{B}\cdot\mathrm{S}, \quad \mathrm{ST}+\mathrm{B} \underset{\mathrm{k_3^{off}}}{\overset{\mathrm{k_3^{on}}}{\rightleftharpoons}} \mathrm{B}\cdot\mathrm{ST} \qquad (\mathrm{S1}) \\
\mathrm{B}\cdot\mathrm{ST} \xrightarrow{\mathrm{k_{dps}}} \mathrm{B}\cdot\mathrm{S} \xrightarrow{\mathrm{k_{dps}}} \mathrm{B}\cdot\mathrm{U} \\
\mathrm{ST} \xrightarrow{\mathrm{k_{dps}}} \mathrm{S} \xrightarrow{\mathrm{k_{dps}}} \mathrm{U} \\
\mathrm{ST} \xrightarrow{\mathrm{k_{dps}}} \mathrm{T} \xrightarrow{\mathrm{k_{dps}}} \mathrm{U}.
\end{gathered}
$$

The corresponding mass actions equations are:

$$\frac{\mathrm{d}[U]}{\mathrm{d}t} = k_1^b[AU] - k_1^f[A][U] + k_1^{off}[BU] - k_1^{on}[U][B] - k_{ps}[U] + k_{dps}([S]+[T]), \tag{S1}$$

$$\frac{\mathrm{d}[AU]}{\mathrm{d}t} = k_1^f[A][U] - (k_{cat}+k_1^b)[AU], \tag{S2}$$

$$\frac{\mathrm{d}[T]}{\mathrm{d}t} = k_{cat}[AU] + k_2^b[AT] + k_{ps}([U]-[T]) - k_2^f[A][T] + k_{dps}([ST]-[T]), \tag{S3}$$

$$\frac{\mathrm{d}[AT]}{\mathrm{d}t} = k_2^f[A][T] - k_2^b[AT] - k_{cat}[AT], \tag{S4}$$

$$\frac{\mathrm{d}[ST]}{\mathrm{d}t} = k_{ps}[T] + k_3^{off}[BST] - k_3^{on}[ST][B] + k_{cat}[AT] - k_{dps}[ST], \tag{S5}$$

$$\frac{\mathrm{d}[BST]}{\mathrm{d}t} = -k_{dps}[BST] - k_3^{off}[BST] + k_3^{on}[ST][B], \tag{S6}$$

$$\frac{\mathrm{d}[BS]}{\mathrm{d}t} = k_{dps}[BST] - k_2^{off}[BS] + k_2^{on}[S][B] - k_{dps}[BS], \tag{S7}$$

$$\frac{\mathrm{d}[BU]}{\mathrm{d}t} = k_{dps}[BS] - k_1^{off}[BU] + k_1^{on}[U][B]. \tag{S8}$$

The KaiBC complex sequesters KaiA when the S site is phosphorylated on KaiC (BS and BST), where $n = 2$ indicates the strength of sequestration.

$$[B] = [B]_T - [BU] - [BS] - [BST] \tag{S9}$$

$$[A] = \max\{0, [A]_T - n\cdot([BST]+[BS]) - ([AU]+[AT])\} \quad n = 2 \tag{S10}$$

To study the stability of our model under ATP variations, we adopt the competitive inhibition

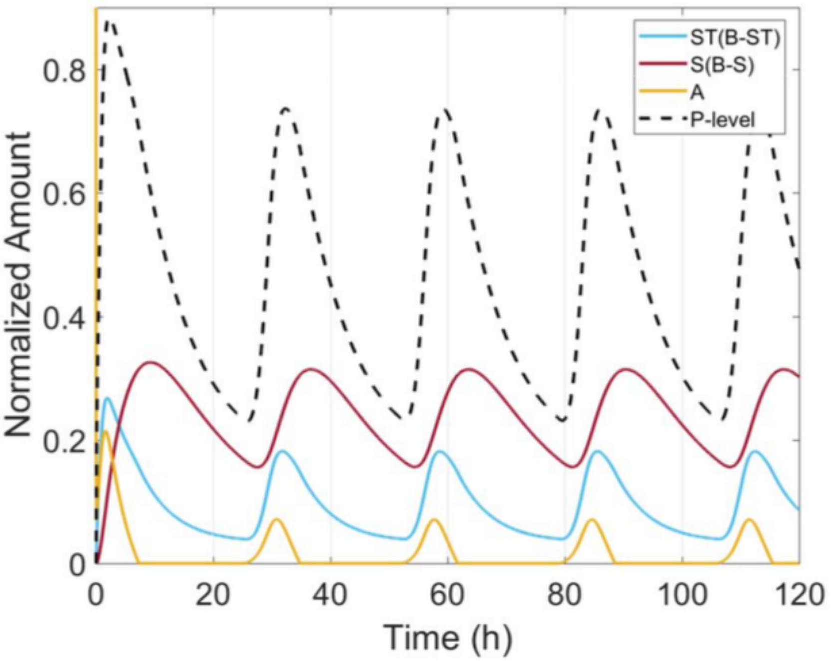

Figure S1: **Simulation of the detailed model.** Oscillations of KaiA (Yellow), KaiBC complexes in different stages (Blue and Magenta) along with the overall phosphorylation level (Black). The proportion of phosphorylated KaiC oscillates roughly between 73% and 25% on a circadian period. KaiA is mostly sequestered by KaiBC complexes (mainly by B-S) except for a short phase, which correspond to a rapid rise in the phosphorylation level. See Table S1 for detailed description of the parameters.

among ATP, ADP, and KaiC as in [12].

$$k_{ps} = \frac{[ATP]}{[ATP] + K_I[ADP]} k_{ps}^0 \tag{S11}$$

$$k_{cat} = \frac{[ATP]}{[ATP] + K_I[ADP]} k_{cat}^0 \tag{S12}$$

Here, the parameters k_{ps}^0 and k_{cat}^0 are the constant rates under 100% ATP, and K_I is the strength of the inhibition from ADP. Simulations of the model under various [ATP]/([ATP]+[ADP]) ratios show that the cyanobacterial clock is robust to ATP variations (Figure 2E-F, main text), which is consistent with previous experimental results [12].

2 Bifurcation analysis and stochastic simulations

Next, we generate a bifurcation diagram for the detailed model using XPP-AUTO. Our bifurcation diagram is similar to that in [13]. In particular, oscillations occur when the ratio between the concentration of KaiA and KaiC is within a bounded range while the relative concentration of KaiB has little effect on whether there are oscillations (Figure S2).

We also simulated our model with the Gillespie algorithm (kinetic Monte Carlo). By keeping the KaiC total concentration a constant $C_T = 1\mu M$, the number of KaiC molecules is directly related to the total volume in our simulation. We simulate the discrete model for various C_T and compare the corresponding phosphorylation levels of KaiC (Figure S3). When the number of molecules is low ($C_T = 10, 20, 50$), the trajectory exhibits randomness without any sustained oscillations (Figure S3). As the number of KaiC molecules increases, however, the corresponding oscillations become more stable. When the number of molecules C_T becomes large enough, the general shape of the profile stays almost the same even if the number is doubled from $C_T = 5000$ to $C_T = 10000$. The amplitude of the oscillations from stochastic simulations approaches that of the deterministic model as the number of molecules increases. Moreover, the phase difference between the stochastic and deterministic simulations decreases significantly as the number of molecules increases. Therefore, we predict that to observe stable and synchronized oscillations, the total amount of KaiC proteins must be above a certain threshold.

3 Linear stability analysis of the core model

Here, we present a linear stability analysis of the simplified model. We continue to use $[T]$, $[ST]$, and $[S]$ as the state variables for KaiC concentrations in various phosphorylation states, and the variable $[A]$ for the concentration of KaiA. Constants C_T and A_T indicate the total amount of the KaiC and KaiA proteins, respectively, and K_d is the dissociation constant in the sequestration of KaiA through KaiC-S. We assume that sequestration happens on a faster timescale, thus reaching equilibrium quickly. Due to the two time scales and the binding of KaiA having no impact on other reactions of S, we solve for the concentration of free KaiA.

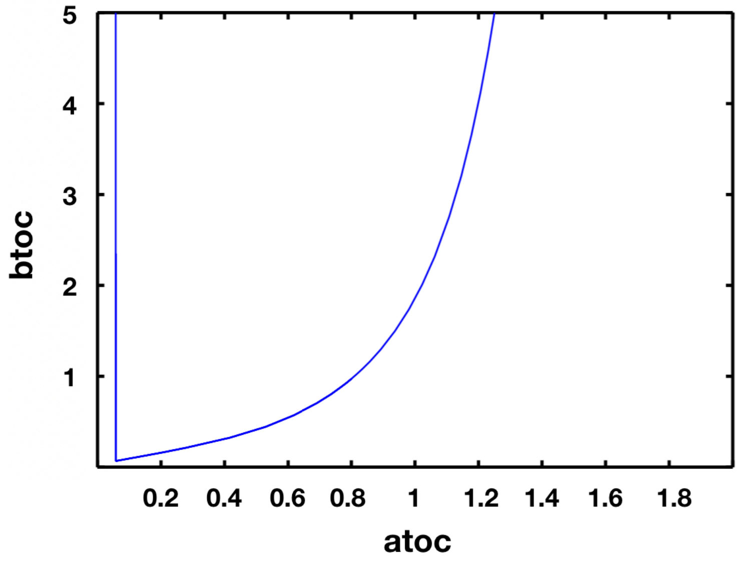

Figure S2: **Two parameter bifurcation diagram of the KaiC system.**. The parameter *atoc* is the fraction of KaiA concentration to KaiC concentration. The parameter *btoc* is the fraction of KaiB concentration to KaiC concentration. Points within the region enclosed by the blue curve give initial distributions that produce oscillations while we expect no oscillations outside this region.

$$\begin{aligned}
[S_{free}] &= K_d[AS] \\
\implies (A_T - [AS])([S] - [AS]) &= K_d[AS] \\
\implies [AS] &= \left(A_T + [S] + K_d - \sqrt{(A_T + [S] + K_d)^2 - 4A_T[S]}\right)/2 \\
\implies [A] = A_T - [AS] &= \left(A_T - [S] - K_d + \sqrt{(A_T - [S] - K_d)^2 + 4K_dA_T}\right)/2.
\end{aligned} \tag{S13}$$

First, we reduce the number of variables by applying the following conservation law

$$[U] = C_T - [T] - [ST] - [S]. \tag{S14}$$

After applying Equation (S14), the core model reduces to the following set of equations.

$$\frac{\mathrm{d}[T]}{\mathrm{d}t} = k_1[A](C_T - [T] - [ST] - [S]) - k_2[T] \tag{S15}$$

$$\frac{\mathrm{d}[ST]}{\mathrm{d}t} = k_2[T] - k_3[ST] \tag{S16}$$

$$\frac{\mathrm{d}[S]}{\mathrm{d}t} = k_3[ST] - k_4[S] \tag{S17}$$

$$[A] = \left(A_T - [S] - K_d + \sqrt{(A_T - [S] - K_d)^2 + 4K_dA_T}\right)/2. \tag{S18}$$

K_d must be small enough for the system to generate sustainable oscillations. Therefore, since $K_d << A_T$, we take the limit as $K_d \to 0$ in $[A]$ to get an approximate expression $[A] = \max\{A_T -$

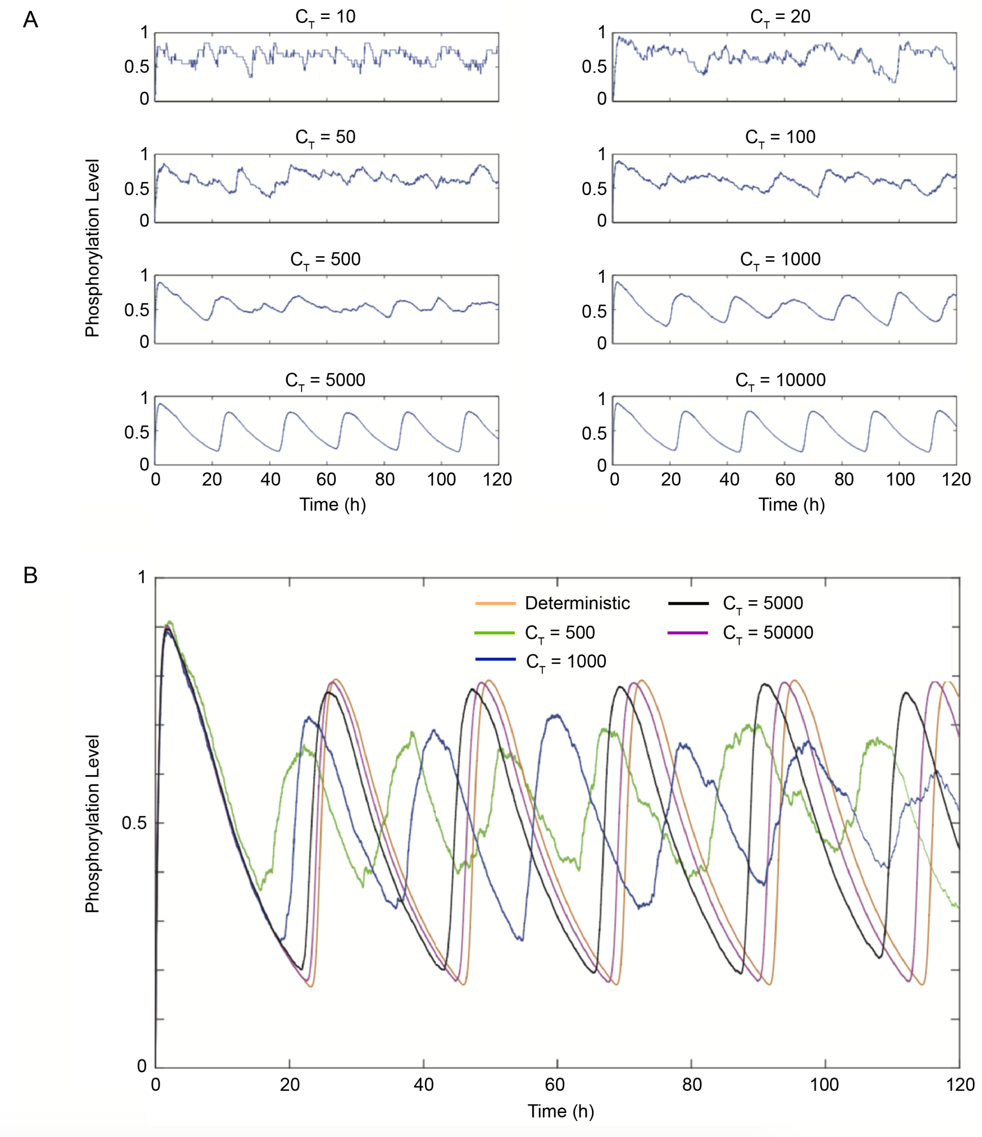

Figure S3: **Stochastic simulations of the detailed model**. (A) Trajectories from stochastic simulations for various levels of total KaiC molecules are compared side by side. The horizontal axis of all plots are 'time (h)' while the vertical axis shows the relative phosphorylation level of KaiC protein. C_T = 10, 20, 50, 100, 500, 1000, 5000, and 10000. (B) Trajectories from stochastic simulations for various levels of total KaiC molecules compared with the deterministic simulation of the detailed model. C_T = 500, 1000, 5000, and 50000.

$[S], 0\}$. Then, Equations (S15)-(S18) reduce to:

$$\frac{\mathrm{d}[T]}{\mathrm{d}t} = k_1(A_T - [S])(C_T - [T] - [ST] - [S]) - k_2[T] \tag{S19}$$

$$\frac{\mathrm{d}[ST]}{\mathrm{d}t} = k_2[T] - k_3[ST] \tag{S20}$$

$$\frac{\mathrm{d}[S]}{\mathrm{d}t} = k_3[ST] - k_4[S]. \tag{S21}$$

Solving the above system for the equilibrium solutions, we obtain $[ST] = \frac{k_4}{k_3}[S]$ and $[T] = \frac{k_4}{k_2}[S]$ from Equation (S20) and Equation (S21), respectively. Solving for the equilibrium solution of Equation (S19), we obtain a quadratic in terms of $[S]$:

$$k_1(A_T - [S])(C_T - (1 + \frac{k_4}{k_2} + \frac{k_4}{k_3})[S]) - k_4[S] = 0. \tag{S22}$$

Additionally, we assume $k_4 << k_1$ since KaiA-enhanced phosphorylation is much faster than (auto)phosphorylation. Hence, we drop the last term in Equation (S22). After dividing k_1 through all terms, we find two steady state solutions:

$$(A_T - [S])(C_T - (1 + \frac{k_4}{k_2} + \frac{k_4}{k_3})[S]) - \frac{k_4}{k_1}[S] = 0 \tag{S23}$$

$$\implies (A_T - [S])(C_T - (1 + \frac{k_4}{k_2} + \frac{k_4}{k_3})[S]) = 0 \tag{S24}$$

$$\implies [S]^* = A_T \quad \text{and} \quad [S]^* = \frac{C_T}{1+r}, \quad \text{where} \quad r = \frac{k_4}{k_2} + \frac{k_4}{k_3}. \tag{S25}$$

Next, we compute the Jacobian at the equilibrium $[S]^* = A_T$:

$$J_1 = \begin{bmatrix} -k_2 & 0 & -k_1(C_T - (1+r)A_T) \\ k_2 & -k_3 & 0 \\ 0 & k_3 & -k_4 \end{bmatrix}.$$

The characteristic function of the Jacobian, J_1, is then:

$$p_1(\lambda) = (\lambda + k_2)(\lambda + k_3)(\lambda + k_4) + k_1k_2k_3(C_T - (1+r)A_T). \tag{S26}$$

From the secant condition [40], we conclude that oscillations occur when

$$\frac{k_1k_2k_3(C_T - (1+r)A_T)}{k_2k_3k_4} \geq (\sec(\frac{\pi}{3}))^3 \tag{S27}$$

$$C_T - (1+r)A_T \geq \frac{8k_4}{k_1}. \tag{S28}$$

In the main text, we write condition (S28) as $C_T - (1+r)A_T \geq \epsilon$, where $r = \frac{k_4}{k_2} + \frac{k_4}{k_3}$ and $\epsilon = \frac{8k_4}{k_1}$. To verify our analysis, we simulate the model for $k_2 = k_3 = k_4 = 0.1$ and compare our results with $C_T - 3A_T > \frac{.8}{k_1}$.

Next, we analyze the Jacobian matrix at the other equilibrium $[S]^* = \frac{C_T}{3}$. Without introducing too many computational details, we assume that $k_2 = k_3 = k_4$, which means that autodephosphorylation and autophosphorylation of KaiC are on the same time scale.

The Jacobian of $[S]^* = \frac{C_T}{3}$ is:

$$J_2 = \begin{bmatrix} -k_1(A_T - \frac{C_T}{3}) - k_2 & -k_1(A_T - \frac{C_T}{3}) & -k_1(A_T - \frac{C_T}{3}) \\ k_2 & -k_2 & 0 \\ 0 & k_2 & -k_2 \end{bmatrix}.$$

The characteristic polynomial of the Jacobian, J_2, is a cubic function:

$$\begin{aligned} p_2(\lambda) &= (b + k_2 + \lambda)(k_2 + \lambda)^2 + bk_2(k_2 + \lambda) + bk_2^2 \\ &= (\lambda + k_2)^3 + b(k_2 + \lambda)^2 + bk_2(k_2 + \lambda) + bk_2^2 \\ &= \lambda^3 + (3k_2 + b)\lambda^2 + (3k_2^2 + 3bk_2)\lambda + k_2^3 + 3bk_2^2, \end{aligned} \tag{S29}$$

where $b = k_1(A_T - \frac{C_T}{3}) > 0$.

The Routh-Hurwitz Stability Criterion [41] states that a necessary condition for a cubic function $h(x) = x^3 + a_2x^2 + a_1x + a_0$ to be stable (i.e., all roots have negative real part) is $a_0 > 0$, $a_2 > 0$, and $a_2a_1 > a_0$.

Applying the criterion to Equation (S29), we have $a_0 > 0$ and $a_2 > 0$. We check the third condition accordingly:

$$\begin{aligned} a_1a_2 - a_0 &= (3k_2 + b)(3k_2^2 + 3bk_2) - k_2^3 - 3bk_2^2 \\ \implies \frac{a_1a_2 - a_0}{k_2} &= (3k_2 + b)(3k_2 + 3b) - k_2^2 - 3bk_2 \\ &= 8k_2^2 + 9bk_2 + 3b^2 > 0, \quad \text{for all } b, k_2 > 0. \end{aligned} \tag{S30}$$

Thus, we know that all three roots of the characteristic function always have negative real parts, implying that the steady state is stable. Therefore, there are no oscillations around the steady state $[S]^* = \frac{C_T}{3}$.

To conclude, we found a necessary condition for the system to generate oscillations, which we also verified by simulations.

A

Description	Symbol	Value
Binding rate between KaiA and unphosphorylated KaiC (U)	k_1^f	20.73 /μMhr
Binding rate between KaiA and T-phosphorylated KaiC	k_2^f	87.59 /μMhr
Unbinding rate between KaiA and unphosphorylated KaiC (U)	k_1^b	3.338 /hr
Unbinding rate between KaiA and T-phosphorylated KaiC (T)	k_2^b	6.113 /hr
Binding rate between KaiB and unphosphorylated KaiC (U)	k_1^{on}	11.50 /μMhr
Binding rate between KaiB and KaiC-S	k_2^{on}	66.79 /μMhr
Binding rate between KaiA and doubly-phosphorylated KaiC (ST)	k_3^{on}	7.18 /μMhr
Unbinding rate between KaiB and unphosphorylated KaiC (U)	k_1^{off}	10.72 /hr
Unbinding rate between KaiB and KaiC-S	k_2^{off}	12.32 /hr
Unbinding rate between KaiA and doubly-phosphorylated KaiC (ST)	k_3^{off}	13.59 /hr
KaiA-enhanced phosphorylated of KaiC	k_{cat}	28.15 /hr
KaiC autophosphorylation rate	k_{ps}	.0384 /hr
KaiC autodephosphorylation rate	k_{dps}	.1270 /hr
Total KaiC concentration	C_T	1 μM
Total KaiA concentration	A_T	1.0401 μM
Total KaiB concentration	B_T	1.3468 μM

B

Description	Symbol	Value
Phosphorylation rate from T to ST	k_2	.1/hr
Dephosphorylation rate from ST to S	k_3	.1/hr
Dephosphorylation rate from S to U	k_4	.1 /hr
Transcription rate (constitutive)	V_{trsp}	.05 $\mu M/hr$
KaiC degradation rate	V_d	.05 /hr
mRNA degradation rate	V_m	.1 /hr
Adjustment parameter	K_0	1 μM
**Parameters perturbed for robustness**	**Symbol**	**Range**
KaiA-enhaned phosphorylation rate of unphosphorylated U to T	k_1	$0.1 \sim 25/\mu$Mhr
Dissociation between KaiA and S	K_d	$10^{-4} \sim 10^{-1}\mu$M
Total KaiC concentration	C_T	$0.01 \sim 30\mu$M
Total KaiA concentration	A_T	$0.01 \sim 10\mu$M
Translation rate	K_s	$0.01 \sim 10$/hr

Table S1: Description of parameters used in simulations for (A) the detailed mathematical model and (B) the core, TTFL, and PTR models.

4 Derivation of the repression function in the novel phospholock model

From Figure 4A in the main text, we have the following reaction network.

$$\begin{aligned} \mathrm{A} + \mathrm{R} &\underset{\mathrm{k}_{1r}}{\overset{\mathrm{k}_{1f}}{\rightleftharpoons}} \mathrm{AR} \\ \mathrm{AR} &\underset{\mathrm{k}_{2r}}{\overset{\mathrm{k}_{2f}}{\rightleftharpoons}} \mathrm{ARp} \\ \mathrm{ARp} &\xrightarrow{\mathrm{k}_3} \mathrm{A} + \mathrm{Rp} \end{aligned} \tag{S31}$$

Network (S31) generates the following system of ODEs.

$$\begin{aligned} \frac{\mathrm{dA}}{\mathrm{d}t} &= k_{1r}\mathrm{AR} + k_3\mathrm{ARp} - k_{1f}[\mathrm{A}][\mathrm{R}] \\ \frac{\mathrm{dAR}}{\mathrm{d}t} &= k_{1f}[\mathrm{A}][\mathrm{R}] + k_{2r}\mathrm{ARp} - (k_{1r} + k_{2f})\mathrm{AR} \\ \frac{\mathrm{dARp}}{\mathrm{d}t} &= k_{2f}\mathrm{AR} - (k_{2r} + k_3)\mathrm{ARp} \end{aligned} \tag{S32}$$

System (S32) generates the following steady-state equations.

$$k_{1r}\mathrm{AR} + k_3\mathrm{ARp} = k_{1f}[\mathrm{A}][\mathrm{R}] \tag{S33}$$

$$k_{1f}[\mathrm{A}][\mathrm{R}] + k_{2r}\mathrm{ARp} = (k_{1r} + k_{2f})\mathrm{AR} \tag{S34}$$

$$k_{2f}\mathrm{AR} = (k_{2r} + k_3)\mathrm{ARp} \tag{S35}$$

Additionally, we have the following two conservation laws in total activator and total repressor concentrations.

$$A_T = \mathrm{A} + \mathrm{AR} + \mathrm{ARp} \tag{S36}$$

$$R_T = \mathrm{R} + \mathrm{AR} + \mathrm{ARp}, \tag{S37}$$

where A_T denotes the total concentration of A and R_T denotes the total concentration of R.

Using Eqn. (S35), we get

$$\mathrm{ARp} = \frac{k_{2f}}{k_{2r} + k_3}\mathrm{AR}. \tag{S38}$$

Combining the conservation law (S36) with Eqn. (S38), we get an updated conservation laws

$$A_T = \mathrm{A} + \left(1 + \frac{k_{2f}}{k_{2r} + k_3}\right)\mathrm{AR} \tag{S39}$$

$$R_T = \mathrm{R} + \left(1 + \frac{k_{2f}}{k_{2r} + k_3}\right)\mathrm{AR}. \tag{S40}$$

Moreover, substituting ARp based on Eqn. (S35) into the steady-state equation (S33) gives

$$k_{1r}\mathrm{AR} + \frac{k_{2f}k_3}{k_{2r}+k_3}\mathrm{AR} = k_{1f}[\mathrm{A}][\mathrm{R}],$$

or

$$\left(K_d + \frac{k_{2f}k_3}{k_{1f}(k_{2r}+k_3)}\right)\mathrm{AR} = [\mathrm{A}][\mathrm{R}]. \tag{S41}$$

For simplification, we set

$$\tilde{K}_1 := 1 + \frac{k_{2f}}{k_{2r}+k_3}, \tag{S42}$$

and

$$\tilde{K}_2 := K_d + \frac{k_{2f}k_3}{k_{1f}(k_{2r}+k_3)}. \tag{S43}$$

Then, we can rewrite the conservation laws as

$$\mathrm{A} = A_T - \tilde{K}_1\mathrm{AR} \tag{S44}$$

$$\mathrm{R} = R_T - \tilde{K}_1\mathrm{AR}. \tag{S45}$$

Substituting the conservation laws (S44) and (S45) into Eqn. (S41) gives the quadratic equation

$$\tilde{K}_2\mathrm{AR} = (A_T - \tilde{K}_1\mathrm{AR})(R_T - \tilde{K}_1\mathrm{AR}). \tag{S46}$$

Expanding out Eqn. (S46) gives

$$A_T R_T - (\tilde{K}_1 A_T + \tilde{K}_1 R_T + \tilde{K}_2)\mathrm{AR} + \tilde{K}_1^2\mathrm{AR}^2 = 0. \tag{S47}$$

The solution of Eqn. (S47) is

$$\mathrm{AR} = \frac{\tilde{K}_1 A_T + \tilde{K}_1 R_T + \tilde{K}_2 \pm \sqrt{(\tilde{K}_1 A_T + \tilde{K}_1 R_T + \tilde{K}_2)^2 - 4\tilde{K}_1^2 A_T R_T}}{2\tilde{K}_1^2}.$$

We take the negative solution above to get that

$$\mathrm{AR} = \frac{\tilde{K}_1 A_T + \tilde{K}_1 R_T + \tilde{K}_2 - \sqrt{(\tilde{K}_1 A_T + \tilde{K}_1 R_T + \tilde{K}_2)^2 - 4\tilde{K}_1^2 A_T R_T}}{2\tilde{K}_1^2}. \tag{S48}$$

Finally, we replace the solution for AR in Eqn. (S48) into the conservation law (S44) to get the

concentration of free activator.

$$
\begin{aligned}
&\mathrm{A} = A_T - \tilde{K}_1\mathrm{AR}\\
&\implies \mathrm{A} = A_T - \frac{\tilde{K}_1 A_T + \tilde{K}_1 R_T + \tilde{K}_2 - \sqrt{(\tilde{K}_1 A_T + \tilde{K}_1 R_T + \tilde{K}_2)^2 - 4\tilde{K}_1^2 A_T R_T}}{2\tilde{K}_1}\\
&\implies \mathrm{A} = \frac{2\tilde{K}_1 A_T}{2\tilde{K}_1} - \frac{\tilde{K}_1 A_T + \tilde{K}_1 R_T + \tilde{K}_2 - \sqrt{(\tilde{K}_1 A_T + \tilde{K}_1 R_T + \tilde{K}_2)^2 - 4\tilde{K}_1^2 A_T R_T}}{2\tilde{K}_1}\\
&\implies \boxed{\mathrm{A} = \frac{\tilde{K}_1 A_T - \tilde{K}_1 R_T - \tilde{K}_2 + \sqrt{(\tilde{K}_1 A_T + \tilde{K}_1 R_T + \tilde{K}_2)^2 - 4\tilde{K}_1^2 A_T R_T}}{2\tilde{K}_1}.}
\end{aligned}
$$

In the main text, we replace R_T with R as done previously [14, 16].

5 Derivation of the repression function in the adjusted phospholock model with activator phosphorylation

From Figure 4D in the main text, we have the following reaction network.

$$
\begin{aligned}
\mathrm{A} + \mathrm{R} &\underset{\mathrm{k}_{1r}}{\overset{\mathrm{k}_{1f}}{\rightleftharpoons}} \mathrm{AR}\\
\mathrm{AR} &\underset{\mathrm{k}_{2r}}{\overset{\mathrm{k}_{2f}}{\rightleftharpoons}} \mathrm{ApRp}\\
\mathrm{ApRp} &\xrightarrow{\mathrm{k}_3} \mathrm{Ap} + \mathrm{Rp}\\
\mathrm{Ap} &\underset{\mathrm{k}_{4f}}{\overset{\mathrm{k}_{4r}}{\rightleftharpoons}} \mathrm{A}.
\end{aligned}
\tag{S49}
$$

Network (S49) generates the following system of ODEs.

$$
\begin{aligned}
\frac{\mathrm{dA}}{\mathrm{d}t} &= k_{1r}\mathrm{AR} + k_{4r}\mathrm{Ap} - k_{1f}[\mathrm{A}][\mathrm{R}] - k_{4f}\mathrm{A}\\
\frac{\mathrm{dAR}}{\mathrm{d}t} &= k_{1f}[\mathrm{A}][\mathrm{R}] + k_{2r}\mathrm{ApRp} - (k_{1r} + k_{2f})\mathrm{AR}\\
\frac{\mathrm{dApRp}}{\mathrm{d}t} &= k_{2f}\mathrm{AR} - (k_{2r} + k_3)\mathrm{ApRp}\\
\frac{\mathrm{dAp}}{\mathrm{d}t} &= k_3\mathrm{ApRp} + k_{4f}\mathrm{A} - k_{4r}\mathrm{Ap}
\end{aligned}
\tag{S50}
$$

System (S50) generates the following steady-state equations.

$$k_{1r}\mathrm{AR} + k_{4r}\mathrm{Ap} = k_{1f}[\mathrm{A}][\mathrm{R}] + k_{4f}\mathrm{A} \tag{S51}$$

$$k_{1f}[\mathrm{A}][\mathrm{R}] + k_{2r}\mathrm{ApRp} = (k_{1r} + k_{2f})\mathrm{AR} \tag{S52}$$

$$k_{2f}\mathrm{AR} = (k_{2r} + k_3)\mathrm{ApRp} \tag{S53}$$

$$k_3\mathrm{ApRp} + k_{4f}\mathrm{A} = k_{4r}\mathrm{Ap} \tag{S54}$$

Additionally, we have the following two conservation laws in total activator and total repressor concentrations.

$$A_T = \mathrm{A} + \mathrm{AR} + \mathrm{ApRp} + \mathrm{Ap} \tag{S55}$$

$$R_T = \mathrm{R} + \mathrm{AR} + \mathrm{ApRp}, \tag{S56}$$

where A_T denotes the total concentration of A and R_T denotes the total concentration of R.

Using Eqn. (S53), we get

$$\mathrm{AR} = \frac{k_{2f}}{k_{2r} + k_3}\mathrm{ApRp}. \tag{S57}$$

Moreover, Eqn. (S54) gives

$$\begin{aligned} \mathrm{Ap} &= \frac{k_3}{k_{4r}}\mathrm{ApRp} + \frac{k_{4f}}{k_{4r}}\mathrm{A} \\ \implies \mathrm{Ap} &= \frac{k_{2f}k_3}{k_{4r}(k_{2r} + k_3)}\mathrm{AR} + k_4\mathrm{A}, \end{aligned} \tag{S58}$$

where $k_4 := \frac{k_{4f}}{k_{4r}}$.

Next, combining the conservation law (S55) with Eqns. (S57) and (S58) gives updated conservation laws

$$A_T = (1 + k_4)\mathrm{A} + \left(1 + \frac{k_{2f}}{k_{2r} + k_3} + \frac{k_{2f}k_3}{k_{4r}(k_{2r} + k_3)}\right)\mathrm{AR} \tag{S59}$$

$$R_T = \mathrm{R} + \tilde{K}_1\mathrm{AR}, \tag{S60}$$

where $\tilde{K}_1$ is defined as in (S42).

Moreover, substituting Ap based on Eqn. (S58) into the steady-state equation (S51) gives

$$\tilde{K}_2\mathrm{AR} = [\mathrm{A}][\mathrm{R}], \tag{S61}$$

where $\tilde{K}_2$ is defined as in (S43). For simplification, we set

$$\tilde{K}_3 := \tilde{K}_1 + \frac{k_{2f}k_3}{k_{4r}(k_{2r} + k_3)}. \tag{S62}$$

Then, we can rewrite the conservation laws as

$$(1 + k_4)\mathrm{A} = A_T - \tilde{K}_3\mathrm{AR} \tag{S63}$$

$$\mathrm{R} = R_T - \tilde{K}_1\mathrm{AR}. \tag{S64}$$

Substituting the conservation laws (S63) and (S64) into Eqn. (S61) gives the quadratic equation

$$\tilde{K}_2\mathrm{AR} = \frac{(A_T - \tilde{K}_3\mathrm{AR})(R_T - \tilde{K}_1\mathrm{AR})}{1 + k_4}. \tag{S65}$$

Expanding out Eqn. (S65) gives

$$A_T R_T - (\tilde{K}_1 A_T + \tilde{K}_3 R_T + \tilde{K}_2(1 + k_4))\text{AR} + \tilde{K}_1 \tilde{K}_3 \text{AR}^2 = 0. \tag{S66}$$

The solution of Eqn. (S66) is

$$\text{AR} = \frac{\tilde{K}_1 A_T + \tilde{K}_3 R_T + \tilde{K}_2(1 + k_4) \pm \sqrt{(\tilde{K}_1 A_T + \tilde{K}_3 R_T + \tilde{K}_2(1 + k_4))^2 - 4\tilde{K}_1 \tilde{K}_3 A_T R_T}}{2\tilde{K}_1 \tilde{K}_3}.$$

We take the negative solution above to get that

$$\text{AR} = \frac{\tilde{K}_1 A_T + \tilde{K}_3 R_T + \tilde{K}_2(1 + k_4) - \sqrt{(\tilde{K}_1 A_T + \tilde{K}_3 R_T + \tilde{K}_2(1 + k_4))^2 - 4\tilde{K}_1 \tilde{K}_3 A_T R_T}}{2\tilde{K}_1 \tilde{K}_3}. \tag{S67}$$

Finally, we replace the solution for AR in Eqn. (S67) into the conservation law (S63) to get the concentration of free activator.

$$\begin{aligned}
&(1 + k_4)\text{A} = A_T - \tilde{K}_3 \text{AR} \\
\implies &(1 + k_4)\text{A} = A_T - \frac{\tilde{K}_1 A_T + \tilde{K}_3 R_T + \tilde{K}_2(1 + k_4) - \sqrt{(\tilde{K}_1 A_T + \tilde{K}_3 R_T + \tilde{K}_2(1 + k_4))^2 - 4\tilde{K}_1 \tilde{K}_3 A_T R_T}}{2\tilde{K}_1} \\
\implies &(1 + k_4)\text{A} = \frac{2\tilde{K}_1 A_T}{2\tilde{K}_1} - \frac{\tilde{K}_1 A_T + \tilde{K}_3 R_T + \tilde{K}_2(1 + k_4) - \sqrt{(\tilde{K}_1 A_T + \tilde{K}_3 R_T + \tilde{K}_2(1 + k_4))^2 - 4\tilde{K}_1 \tilde{K}_3 A_T R_T}}{2\tilde{K}_1} \\
\implies &\boxed{\text{A} = \frac{\tilde{K}_1 A_T - \tilde{K}_3 R_T - \tilde{K}_2(1 + k_4) + \sqrt{(\tilde{K}_1 A_T + \tilde{K}_3 R_T + \tilde{K}_2(1 + k_4))^2 - 4\tilde{K}_1 \tilde{K}_3 A_T R_T}}{2\tilde{K}_1(1 + k_4)}.}
\end{aligned} \tag{S68}$$

6 Sensitivity of transcription-rate function decreases with increasing k_3 values

Additionally, we performed a comprehensive analysis of how the peak in sensitivity varies with respect to k_3. Previous studies illustrated that a higher sensitivity increases the likelihood of oscillations [14]. We saw for individual parameter sets, for which System (N) exhibited oscillations, that the magnitude of the peak sensitivity decreases as k_3 increases (Figure S4A). Thus, we further investigated whether this is true in general. In particular, for 1000 randomly generated parameter sets for which System (N) exhibited oscillations, we calculate the magnitude of the peak sensitivity first at the k_3 value of the parameter set. Then, we vary k_3 values (x-axis, Figure S4B) and report that variation as percent change from the original k_3 value of the parameter set. Next, we compute the magnitudes of the peak sensitivity for the varied k_3 values and report those as percent variation from the original magnitude (y-axis, Figure S4B). Thus, at 100%, which is the original k_3 value, the magnitude should be 100%, i.e., equal to the original amount. As k_3 increases relative to the

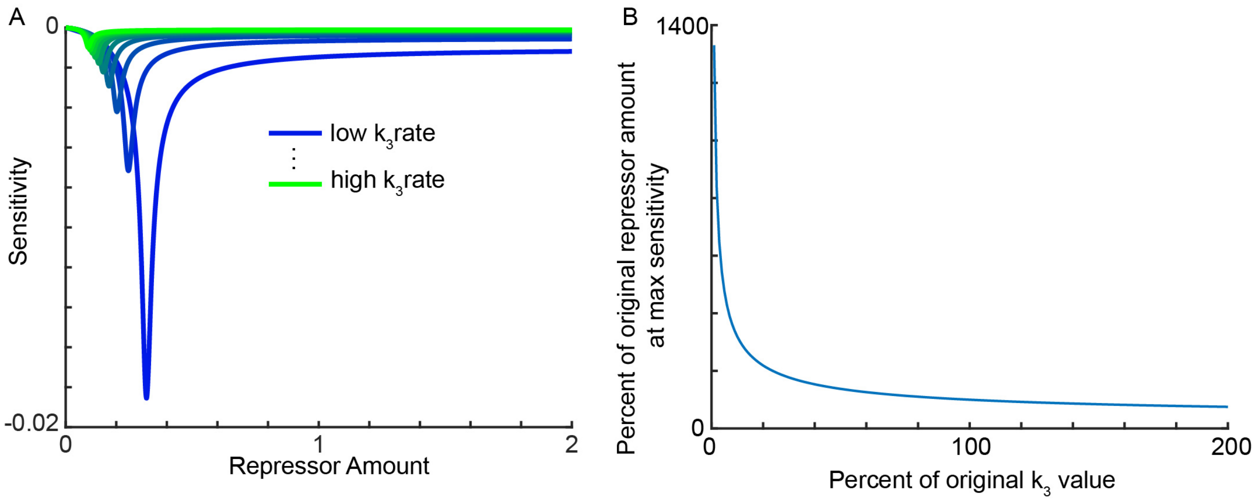

Figure S4: **The robustness of oscillations decreases as the rate of dissocation increases.**(A) The sensitivity of the transcription-rate function of System (N) with one parameter set for which the system exhibited oscillations. We vary the k_3 value to represent an increase in the dissociation rate of phosphorylated repressors. As k_3 increases (more green), the magnitude of the peak sensitivity decreases, i.e., the robustness of oscillations decreases. (B) We randomly generated 1000 parameter sets for which the Neurospora system exhibits oscillations. For each parameter set, we vary the k_3 value relative to the original k_3 value of the parameter set (x-axis records the percent variation). Then, we plot the variation in the magnitude of the peak sensitivity. We compute this variation for each parameter set and plot the mean change. As k_3 increases relative to the original value, the magnitude of the peak sensitivity decreases, indicating that faster dissociation of phosphorylated repressor and activator decreases the robustness of the oscillations.

original k_3 value, the magnitude in the peak sensitivity decreases relative to the original magnitude (Figure S4B). Similarly, when the k_3 value is higher than the original value, the magnitude is also higher than the original amount (Figure S4B). Thus, in general, as the rate of dissociation increases, the magnitude of the peak sensitivity decreases. Therefore, increasing the dissociation rate of the phosphorylated repressor and activator decreases the robustness of the oscillations. This phenomenon may explain why CRY1 binds with BMAL1/CLOCK on DNA independently of PER in the early morning [41].
