# Supplementary material for: Evolution of the repression mechanisms in circadian clocks
Source: Genome Biol. 2022 Jan 10;23:17. doi: 10.1186/s13059-021-02571-0 (PMC8751359; doi:10.1186/s13059-021-02571-0)
Supplement: Supplementary file 2 — Additional file 2 Review history. [file 13059_2021_2571_MOESM2_ESM.docx]

**Review history**

**First round of review**

**Reviewer 1**

In the submitted manuscript, the authors constructed six mathematical models to investigate significance of transcriptional mechanisms in cyanobacteria and eukaryotic clocks. Previously, they proposed a protein sequestration model as a repression mechanism for the negative feedback loop of circadian machinery. In this work, they integrated the protein sequestration model, phosphorylation, and transcriptional mechanisms to compare and contrast cyanobacteria and eukaryotic clocks demonstrating that: 1) phosphorylation increases the robustness of the oscillations and 2) additional phosphorylation of the activator relaxes stoichiometric ratio requirement. The design of the study is well-constructed and the results provide an interesting perspective comparing cyanobacteria and eukaryotic clocks. There are numerous minor mistakes in the described equations which were concerning.

# Major comments:

**Main text**

- Please provide a brief summary of the cyanobacteria post-translational mechanism that generates autonomous oscillations in the main text.
- Please explicitly describe the difference between TTFL and PTR in the text (Figure 1).
- Page 2: It would be helpful to show profiles of [A] at different values of Kd to show how the value of Kd changes nonlinearity of that function ultimately impacting oscillations.
- Page 3, line 50: The authors write “This matches the proposed role of the Rev-erba TTFL in the mammalian clock.” I think some readers will wonder why Rev-erba TTFL instead of PER/CRY TTFL. To provide some context, I suggest the authors to provide a brief background on their previous work and how the additional Rev-erba TTFL helps with stoichiometric balance.
- Page 4: Similar to the comment above, it would be helpful to show profiles of f(R) at different ratios of k2f:k2r to show how this ratio changes nonlinearity with Kd=1. Also, I suggest the authors explore the relationship between Kd and the ratio of k2f:k2r. Specifically, plot Figure 2C with Kd=0.1, 0.01, 0.001, and 0.0001.
- Page 5: I suggest the authors to include more detailed comparison between the models in pages 4 (E) and 5 (N). Also, what are the values of Kd and ratio of k2f:k2r for the model that incorporated additional phosphorylation of the activator? More importantly, what is the rationale for relaxation of stoichiometry requirement when k3 is increased? Also, why is the robustness decreasing as a function of k3?
- The authors write that their model is consistent with “rapid degradation of the phosphorylated repressors is not required for rhythm generation.” However, this point has not clearly articulated in the manuscript.
- Recently, Yi Liu’s group published a paper indicating that the physical interaction between FRQ and CK1 determines the period of circadian rhythms ranging from 19 to 48 hours (Liu et al, Nature Communications, 2019). If the phosphoclock is the core model, then it’s possible that the key parameters determining phosphoclock should be critical in determining the period of circadian rhythms. Please provide period analysis using the ratios of k1f:k1r, k2f:k2r, k3, and k4f:k4r.
- Similar to the above comment, Takao Kondo’s group recently showed that mutations at position 402 of KaiC dramatically changes the period from 15 to 158 hours (Ito-Miwa et al, PNAS, 2020). I think it will be critical for the authors’ model to reproduce this data and uncover any insights based on their phosphoclock model.
- Lastly, I would like the authors to include experimental predictions that could support their models.

# Supplementary Information

- (Page 8) Equation (13), Line 4: According to the calculations, 𝐴_!_ term should not be in the denominator.

(Page 12) In TTFL Equations, units don’t match in the Hill function. Suggest using the hill function with an additional parameter $K_{0} as V_{trsp}\frac{100}{1+{(\frac{[S]}{K_{o}})}^{4}}$

- (Page 17) Equation (63): Should be corrected as:$\left( 1+k_{4} \right)A=A_{T}-\tilde{K}_{3}AR$

# Minor comments:

**Main text**

- (Page 4) Equation E: Representation of 𝑓(𝑅) have mistakes: It should be $2\tilde{K}_{1}$instead of

$2{\tilde{K}_{1}}^{2}$, and 𝑅 should be replaced by $R_{T}$

'

- (Page 5) Equation N: Representation of 𝑓(𝑅) have mistakes: 𝐾$\tilde{K}_{2}$ should be replaced by

𝐾$\tilde{K}_{2}\left( 1+k_{4} \right).$

- (Page 7) Equation (3): Representation of 𝐴𝑅 have mistakes: 𝐾$\tilde{K}_{2}$ should be replaced by

𝐾$\tilde{K}_{2}\left( 1+k_{4} \right)$

- (page 9) Figure 1-D caption should be corrected as “The core model generates oscillations for a ~~higher~~ lower fraction of parameter sets as 𝐾_._ increases from 10^-4^ to 10^-1^.
- Figure 1-D: Better if the y – axis labelling is more specific. (“Fraction of parameter sets”)

# Supplementary Information

- (Page 2) Section 1, Paragraph 1, line 4: … phosphorylation sites on KaiC-Ser-431 (S431) and KaiC-Thr-432 (T432).
- (Page 4) Equation (9): [𝑈𝐵] should be [𝐵𝑈] to be consistent with the notation.
- [(Page 6) Figure (3): Figure E and F] (Units of the ratios does not match in both figures)
- (Page 7) Supp. Figure 3 caption, line 5: (G-F) should be (G-H).
- (Page 8) Equation (13), Line 3 and Line 4: 𝐴_t_ should be replaced by A_T_.
- (Page 9) Supp. Figure 5, Figure A: C_T_ is mentioned as 𝑣 in the figure. (C_T_ ≈ 𝑣, but 𝑣 is not defined	anywhere	and	C_T_ is	used	everywhere	in	the	text	and	in	the	caption.)
- (Page 10) Equation 18: A_t_ should be replaced by A_T_ in the numerator and term A_T_ should not be in the denominator. (Page 8, Equation (13), Line 4)
- (Page 10) Equation 23: In the last term, it should be $\frac{k_{4}}{k_{1}}$ instead of $\frac{k_{4}}{k_{3}}$
- (Page 10) Equation 24: It should be $(1+ \frac{k_{4}}{k_{2}}+ \frac{k_{4}}{k_{3}}$ ) instead of $( \frac{k_{4}}{k_{2}}+ \frac{k_{4}}{k_{3}})$
- (Page 11) Jacobian Matrix 𝐽_2_: Close of brackets missing in the first row, second column term.
- (Page 11) Equation (29), Line 2: Typo in the last term. It should be as: $bk_{2}^{2}$

(Page 11) Equation (30), Line 1 and 2: Last term should be negative instead of positive. (Line 1: $-3bk_{2}^{2}$, Line 2: −3𝑏𝑘_2-_). From line 1 to 2, k_2_ term is factored, therefore line 1 is not equal to line 2 (Suggest using =>).

-

- (Page 12) In TTFL Equations: Equation for [𝐴], term A_T_ should not be in the denominator.
- (Page 13) In PTR Equations: Equation for [𝐴], term A_T_ should not be in the denominator.
- (Page	14)	Table	A,	row	2:	Unit	of $k_{2}^{f}$	should	be 1/𝜇𝑀ℎ𝑟.
- (Page	14)	Table	A, row	8,9,10:	Unit	of $k_{1}^{off},k_{2}^{off}, k_{3}^{off}$ should be 1/ℎ𝑟.

- (Page 14) Table B, row 4: Unit of 𝑉_trsp_ for TTFL with new parameter 𝐾_0_ is 𝜇𝑀/ℎ𝑟. Unit of 𝑉_trsp_ for PTR is 𝜇𝑀/ℎ𝑟.
- (Page 14) Table B, row 8: Unit of K is 𝜇𝑀, not 1/𝜇𝑀.
- (Page 15) Equation (38): Following two typos should be corrected: $AR_{p}=\frac{k_{2f}}{k_{2r}+k_{3}}AR$
- (Page 17) Equation (51): Last term should be positive, not negative. (+𝑘_4f_ A)
- (Page 17) Equation (55) and Equation (56): 𝐴𝑅_p_ should be corrected as 𝐴_p_𝑅_p_.
- (Page 17) Equation (57): Should be corrected as: $A_{p}R_{p}=\frac{k_{2f}}{k_{2r}+k_{3}}AR$
- $\frac{k_{2f}}{k_{2r}+k_{3}}AR\frac{k_{2f}}{k_{2r}+k_{3}}AR$
- (Page 17) Equation (58): Should be corrected as: $A_{p}=\frac{k_{3}}{k_{4r}}A_{p}R_{p}+\frac{k_{4f}}{k_{4r}}A$
- (Page 17) Equation (60): where $\tilde{K}_{1}$is defined as ~~above~~ in (42)
- (Page 18) Equation (65): Should be corrected as: $\tilde{K}_{2}AR=\frac{(A_{T}-\tilde{K}_{3}AR)(R_{T}-\tilde{K}_{1}AR)}{(1+k_{4})}$
- (Page 18) Equations (66) to (68): In all the equations 𝐾$\tilde{K}_{2}$ should be replaced by $\tilde{K}_{2}(1+k_{4})$

**Reviewer 2**

The authors analyze conceptual models of circadian rhythms and extract several interesting features that contribute to self-sustained oscillations beyond the classical Goodwin concept with inhibition via unrealistically high Hill coefficients. I find the following aspects very interesting: balance of activator and inhibitor (following Kim/Forger), fine-tuning of the balance via auxiliary transcriptional feedbacks (proposed also in Kim/Forger), an essential roles of sequestration (discussed by several authors earlier), and of phosphorylation (leading to inactive proteins that could replace degradation as pointed out by Larrondo). Most of these ideas are not really new. In the manuscript, however, interesting mathematical results are presented combined with extensive simulations with randomly selected parameters. I find also the comparative approach (cyanobacteria, Neurospora, mammals) of interest since it indicates that the above principles might represent generic features.

Despite these positive aspects I cannot support the idea to present the results in a Short Report with extensive supplements. The condensation of all the results to a few pages with just 2 figures makes the paper inaccessible to most readers. In my eyes, the manuscript is only understandable to readers familiar with the clocks in cyanobacteria, fungi, and mammals, with the ideas of sequestration, and with previous studies such as the Kim/Forger paper.

Specific comments:

The idea of KaiA sequestration with already described in a modeling papers by Clodong et al. (MSB 2007) and Axmann et al. (Genome Inf. 2007). The replacement of high Hill coefficients has been discussed in a convincing manner already by Gonze (PLoS One 2013). Sequestration as substitute of direct feedbacks has been discussed extensively in papers by S. Legewie and in Jolley et al. (Cell Reports 2012). Inactive complexes in fungi and mammals controlled by phosphorylation have been discussed extensively by Aryal et al. (Mol Cell 2017), Chiou et al. (PNAS 2016), Upadyuy et al. (Sci Reports 2020), Partch (JMB 2020).

I find the explanation of equations (c) insufficient. Only with the aid of Fig.1 and of the Kim/Forger paper readers can follow.

"r is a constant of order one" is confusing.

C_T > 3A might be simpler than introducing epsilon.

Since there are no clear homologies between clock protein in cyanobacteria, fungi, and mammals I suggest to avoid the term evolution. It is more the use of common minimal design principles than a real evolution.

The left part of Fig.2C is not informative.

**Authors’ response to reviewers**

# Reviewer 1:

In the submitted manuscript, the authors constructed six mathematical models to investigate sig- nificance of transcriptional mechanisms in cyanobacteria and eukaryotic clocks. Previously, they proposed a protein sequestration model as a repression mechanism for the negative feedback loop of circadian machinery. In this work, they integrated the protein sequestration model, phosphoryla- tion, and transcriptional mechanisms to compare and contrast cyanobacteria and eukaryotic clocks demonstrating that: 1) phosphorylation increases the robustness of the oscillations and 2) addi- tional phosphorylation of the activator relaxes stoichiometric ratio requirement. The design of the study is well-constructed and the results provide an interesting perspective comparing cyanobac- teria and eukaryotic clocks. There are numerous minor mistakes in the described equations which were concerning.

We thank the reviewer for the summary of our work and for the thorough review that helped us to significantly improve our work. We apologize for the minor mistakes in the equations and have thoroughly checked them for the resubmission.

### Major Comments, Main Text:

1. Please provide a brief summary of the cyanobacteria post-translational mechanism that gen erates autonomous oscillations in the main text.

We have moved the brief summary of the cyanobacterial post-translational mechanism that was in the beginning of the Supplementary Information to the main text. I.e., now in the main text at the beginning of the results section:

#### A detailed model of the cyanobacterial clock

*Cyanobacteria are among the simplest organisms to exhibit circadian oscillationsin cellular components. Proper functioning of the cyanobacterial circadian clockrelies on interactions among three proteins: KaiA, KaiB, and KaiC [6]. Central to the protein interactions are two indispensable phosphorylation sites on KaiC-Ser-431 (S431) and KaiC-Thr-432 (T432). Recently, crystal structural analysis ofthe protein complexes in the KaiABC system revealed a detailed sequestration mechanism of KaiA by KaiBC protein complexes (Figure 1A) and confirmed in silico [7, 8]. First, KaiA acts as an enzyme to enhance the phosphorylation of KaiC on theT432 site and, subsequently, the S431 site. Then, once site S431 is phosphory- lated, KaiC undergoes a conformational change from a pre-hydrolysis state to a post-hydrolysis state creating a hub [9]. Simultaneously, KaiB undergoes a fold-change transition into an ac- tive state that is captured by the post-hydrolysis KaiC on the N-terminal (CI) domain. The KaiBC complex recruits KaiA proteins and prevents KaiC activation on the C-terminal (CII) domain. In other words, KaiBC complexes sequester free KaiA proteins.*

2. Please explicitly describe the difference between TTFL and PTR in the text (Figure 1).

We have moved the description and list of equations for both the TTFL and PTR models from the Supplementary Information to the main text to increase clarity and readability.

#### Additional transcriptional-translational feedback loop adds robustness to oscillations in cyanobacteria

*We also find that an additional transcriptional-translational feedback loop (TTFL) plays a significant role in sustaining robust oscillations. Besides the KaiABC system, the histidine kinases SasA and CikA regulate the output signaling from the post-translational oscillator to the transcriptional activity in the in vivo oscillator. The integrated roles of SasA, CikA, and RpaA together with KaiABC in the cyanobacterial circadian clock were summarized recently [20]. When KaiC phosphorylation reaches its peak, SasA binds to the CI domain of the ST- phosphorylated KaiC [9, 21], autophosphorylates and transfers the phosphate group to RpaA, turning on the transcription factor [22]. During the dephosphorylation phase, when KaiB binds to the CI domain to form the KaiBC complex, SasA is released from KaiC. CikA is then recruited by the KaiBC complex, dephosphorylates RpaA and thus, inhibits the transcription. In other words, the transcription of the kaiBC gene is activated when most of the KaiC proteins are highly phosphorylated and is inhibited when KaiC proteins are mostly in the S state. Therefore, we model the TTFL as an inhibition scheme where S431 phosphorylated KaiC acts like an inhibitor of the kaiBC gene (Figure 1C, System* (TTFL) *below).*

d[*M* ]

d*t*	= *Vtrsp*

d[*U* ]

100

[*S*] 4 − *Vm*[*M* ]

( )

1 +	*K*0

d*t*	= *K_s_*[*M* ] − *k*_1_[*A*][*U* ] + *k*_4_[*S*] − *V_d_*[*U* ]

d[*T* ]

d*t* = *k*_1_[*A*][*U* ] − *k*_2_[*T* ] − *V_d_*[*T* ]

d[*ST* ]

d*t*	= *k*_2_[*T* ] − *k*_3_[*ST* ] − *V_d_*[*ST* ]

d[*S*]

d*t*	= *k*_3_[*ST* ] − *k*_4_[*S*] − *V_d_*[*S*]

(	)

[*A*] =	*A_T_* − [*S*] − *K_d_* +	(*A_T_* − [*S*] − *K_d_*)^2^ + 4*K_d_A_T_	/*2

*The variable M is the amount of mRNA transcribed from the kaiBC gene.*

(TTFL)

*We investigate the role of the transcriptional-translational feedback loop (System (TTFL)) as an additional negative feedback loop in the KaiC system by comparing with a modified system with a constitutive source of transcription (Figure 1D, System* (PTR) *below). See Supp. Table 1 for a detailed description of the parameters and the ranges of parameters used.*

d[*M* ]

d*t*	= *V_trsp_* − *V_m_*[*M* ]

d[*U* ]

d*t*	= *K_s_*[*M* ] − *k*_1_[*A*][*U* ] + *k*_4_[*S*] − *V_d_*[*U* ]

d[*T* ]

d*t* = *k*_1_[*A*][*U* ] − *k*_2_[*T* ] − *V_d_*[*T* ]

d[*ST* ]

d*t*	= *k*_2_[*T* ] − *k*_3_[*ST* ] − *V_d_*[*ST* ]

d[*S*]

d*t*	= *k*_3_[*ST* ] − *k*_4_[*S*] − *V_d_*[*S*]

[*A*] = (*A_T_* − [*S*] − *K_d_* + (*A_T_* − [*S*] − *K_d_*)^2^ + 4*K_d_A_T_* ) */*2

(PTR)

*We simulate both Systems* (TTFL) *and* (PTR) *with 22,500 randomly generated parameter sets varying the transcription rate, K_s_, and the total KaiA concentration, A_T_ . The TTFL model generates circadian oscillations under a wide range of parameters (98.84%, Figure 3E), while the PTR model, without the additional feedback loop, only exhibits oscillations when satisfying Condition (1) (60.33%, Figure 3F). Altogether, our analysis and simulations suggest that the TTFL, as an additional negative feedback loop in the cyanobacterial clock, can help sustain the required molar ratio balance through a homeostatic mechanism.*

3. Page 2: It would be helpful to show profiles of [A] at different values of *K_d_* to show how the value of *K_d_* changes nonlinearity of that function ultimately impacting oscillations.

We have added the profiles of [A] based on the last equation in System (C) for various binding affinities as per the suggestion. The added figure can be found in Figure 3A. In addition to the profiles, we added the sensitivity of those curves for the values of *K_d_* as well (Figure 3B). Notice that, as *K_d_* decreases, the cusp of the repression functions sharpens and the magnitude of the maximum sensitivity value increases, indicating that oscillations are more likely as *K_d_* decreases. New Figures 3A and B:

4. Page 3, line 50: The authors write “This matches the proposed role of the Rev-erba TTFL in the mammalian clock.” I think some readers will wonder why Rev-erba TTFL instead of PER/CRY TTFL. To provide some context, I suggest the authors to provide a brief back- ground on their previous work and how the additional Rev-erba TTFL helps with stoichio- metric balance.

We added a paragraph in the new Discussion section that describes the association between the Rev-erb*α* TTFL and the additional TTFL in the cyanobacterial clock.

## *Discussion*

*. . . Moreover, our results reveal stoichiometric balancing requirements for oscillations and how these requirements may be satisfied in the three systems. For example, an additional transcriptional-translational feedback loop (TTFL) in cyanobacteria plays a significant role in sustaining robust oscillations by maintaining the required molar ratio (Condition (1)). This additional feedback loop matches the proposed role of the Rev-erbα TTFL in the mammalian clock [14, 19] that acts synergistically with the core feedback loop to maintain the molar ratio between activators and repressors. For example, when the molar ratio is perturbed to greater than 1 (i.e., more repressor), the additional feedback loop promotes activator production, restoring the 1:1 molar ratio required for robust oscillations [19]. Similarly, when the molar ratio is perturbed in the cyanobacterial system to greater than its required range (i.e., more free KaiC), then RpaA is dephosphorylated leading to low transcription of the kaiBC genes and a decrease in free KaiC, restoring the molar ratio required for robust oscillations.*

5. Page 4: Similar to the comment above, it would be helpful to show profiles of f(R) at different ratios of *k*_2_*_f_* : *k*_2_*_r_* to show how this ratio changes nonlinearity with *K_d_* = 1.

We thank the reviewer for this suggestion. We have added a new analysis that plots *f* (*R*) along with the sensitivity of *f* (*R*) for increasing ratios of *k*_2_*_f_* : *k*_2_*_r_* when *K_d_* = 1 for a specific parameter set (new Figure 4B, see below). We see that, as the ratio *k*_2_*_f_* : *k*_2_*_r_* increases, the magnitude of the sensitivity of the *f* (*R*) function increases. This increase in the sensitivity suggests that increasing the ratio *k*_2_*_f_* : *k*_2_*_r_* will make the oscillations appear or make them more robust. We have revised the text accordingly to reflect this change and expand on the implications of the increasing sensitivity.

### A novel “Phospholock” model of the eukaryotic clock

*. . . In Figure 4B, we plot the repression function f* (*R*) *for a specific parameter set where*

*K_d_* = 1 *while varying the ratio of phosphorylation to dephosphorylation (i.e., k*_2_

= *k*2*f ). As*

*k*2*r*

*k*_2_ *increases, the cusp of the repression function sharpens (Figure 4B, top). Moreover, the*

*magnitude of the peak sensitivity increases as k*_2_ *increases (Figure 4B, bottom).*

6. Also, I suggest the authors explore the relationship between *K_d_* and the ratio of *k*_2_*_f_* : *k*_2_*_r_*. Specifically, plot Figure 2C with *K_d_* = 0.1, 0.01, 0.001, and 0.0001.

We have updated the analysis in Figure 4C (see below) to reflect *K_d_* values 0.1, 0.01, 0.001, and 0.0001 as the reviewer suggested. As expected, as the *K_d_* values increased, the number of parameter sets that were generated increased. Moreover, with smaller *K_d_* values, oscillations

in the system were more likely when dephosphorylation was stronger than phosphorylation. However, between *K_d_* = 0*.*01 and *K_d_* = 0*.*1, that switched. That is, for *K_d_* = 0*.*1 and *K_d_* = 1, the system was more likely to generate oscillations as the strength of phosphorylation over dephosphorylation increased. Thus, at more biologically realistic *K_d_* values, the phospholock makes the system more likely to generate oscillations. We have updated Figure 4C and the text accordingly to reflect this further analysis.

### A novel “Phospholock” model of the eukaryotic clock

*. . . Next, we investigate how the interplay between the K_d_ value and the ratio of the strength of phosphorylation to dephosphorylation (k*_2_*) affects the robustness of oscillations. As K_d_ values decrease, the likelihood that the system generates oscillations increases in general (Figure 4C). Moreover, at small K_d_ values, the system is more likely to generate oscillations when dephosphorylation is stronger than phosphorylation, i.e., k*_2_ *<* 1*. However, as K_d_ increases, e.g., K_d_* ≥ 0*.*1*, the system is more likely to generate oscillations when phosphorylation is stronger than dephosphorylation, i.e., k*_2_ *>* 1 *(Figure 4C).*

7. Page 5: I suggest the authors include more detailed comparison between the models in pages 4 (E) and 5 (N). Also, what are the values of *K_d_* and ratio of *k*_2_*_f_* : *k*_2_*_r_* for the model that incorporated additional phosphorylation of the activator? More importantly, what is the rationale for relaxation of stoichiometry requirement when *k*_3_ is increased? Also, why is the robustness decreasing as a function of *k*_3_?
   - What are the values of *K_d_* and the ratio of *k*_2_*_f_* : *k*_2_*_r_* for the model that incorporated additional phosphorylation of the activator?

We are not sure to which simulation the reviewer is referring. We only used the additional phosphorylation of the activator in our analysis for the current Figure 4E. We give the distributions for the parameters that we used for this example in the Methods section (“Stoichiometric Ratio Analysis”).

- More importantly, what is the rationale for relaxation of the stoichiometry requirement when *k*_3_ is increased?

By introducing the phosphorylation of the activator into the system, we effectively intro- duce another mechanism by which the activator can be inactivated. However, instead of the repressor being locked with the activator as the means of inactivation, no repressor is required to inactivate when the activator is phosphorylated. Therefore, there is less repressor required for inactivation in the phospholock with additional phosphorylation of the activator. Therefore, the stoichiometric ratios that generate oscillations may take a wider range of values.

- Also, why is the robustness decreasing as a function of *k*_3_?

The reason the robustness decreases for increasing *k*_3_ is similar to why the phospholock requires stronger phosphorylation with less binding affinity. That is, the key to the phos- pholock generating oscillations is that the activator is tightly connected to the repressor. In pure protein sequestration, this requirement is equivalent to tight binding. In the phospholock, the tight binding can be relaxed but, in its place, the phosphorylation must be strong to keep the repressor and activator associated. At the end of the repres- sion cycle, the association between the activator and repressor cannot be too quick, i.e., the dissociation of the repressor and activator cannot be too strong. This is reflected computationally in the *k*_3_ parameter. As *k*_3_ increases, the locking of the activator and repressor is weakened, resulting in less robust oscillations.

8. The authors write that their model is consistent with “rapid degradation of the phosphory- lated repressors is not required for rhythm generation.” However, this point has not clearly articulated in the manuscript.

We have augmented the paper from a short report to a research article. In so doing, we split the Results and Discussion sections into two. In this way, we were able to expand on certain statements that may have not been clearly articulated previously. For example, we added a paragraph discussing how our results about *k*_3_ connect to previous experimental results showing that rapid degradation of the repressors is not required forr rhythm generation. In particular, we have added the following paragraph.

## *Discussion*

*. . . The phospholock model also revealed that rapid degradation of the repressor is not required for sustained oscillations, confirming previous experimental results [23, 24, 38]. That is, sim- ulations revealed that decreasing the k*_3_ *value, a surrogate marker of the degradation strength of the phosphorylated repressor, increases the sensitivity of the repression function, thereby increasing the robustness of oscillations. Therefore, our results are consistent with the im- portant feature, now shown experimentally in both Neurospora [38] and mice [23], that rapid degradation of the phosphorylated repressors is not required for rhythm generation.*

9. Recently, Yi Liu’s group published a paper indicating that the physical interaction between FRQ and CK1 determines the period of circadian rhythms ranging from 19 to 48 hours (Liu et al, Nature Communications, 2019). If the phosphoclock is the core model, then it’s

possible that the key parameters determining phosphoclock should be critical in determining the period of circadian rhythms. Please provide period analysis using the ratios of *k*_1_*_f_* : *k*_1_*_r_*, *k*_2_*_f_* : *k*_2_*_r_*, *k*_3_, and *k*_4_*_f_* : *k*_4_*_r_*.

We agree with the reviewer that it is important to understand how interactions between proteins yield a wide range of periods. However, the ratios listed by the reviewer do not directly correspond to interaction between FRQ annd CK1. In this way, it is difficult to assess how the interactions may manifest various periods. Moreover, in this manuscript, we are more concerned with the principles behind which oscillations are generated across various species, not necessarily the properties of those oscillations. Therefore, we do not believe that it fits into the story to add a period analysis of our models, although this is an interesting question and should be pursued in the future.

10. Similar to the above comment, Takao Kondo’s group recently showed that mutations at position 402 of KaiC dramatically changes the period from 15 to 158 hours (Ito-Miwa et al, PNAS, 2020). I think it will be critical for the authors’ model to reproduce this data and uncover any insights based on their phosphoclock model.

Please see our response to the previous comment.

11. Lastly, I would like the authors to include experimental predictions that could support their models.

We have added a paragraph to the discussion outlining a potential experimental confirmation of our results.

## *Discussion*

*. . . Follow-up work could consider simulated evolution in silico experiments that may reveal efficient and robust mechanisms for generating robust oscillations across independent species. Such mechanisms could help further delineate how clocks may have evolved. Additionally, the plant molecular circadian clock is much more complex than that found in other organ- isms, at least in its TTFL structure. Further work should explore how sequestration and the mechanisms to balance molar ratios we discuss may play a role in the plant circadian clock. Further experiments could measure or change the molar ratios of activators and repressors to determine how the phospholock mechanism we propose extends the range where sustained oscillations are found.*

### Major Comments, Supplementary Information:

1. (Page 8) Equation (13), Line 4: According to the calculations, *A_T_* term should not be in the denominator.

Corrected. We also corrected all instances where it was incorrectly present in the denominator as per your other comments.

2. (Page 12) In TTFL Equations, units don’t match in the Hill function. Suggest using the hill

function with an additional parameter $K_{0} as V_{trsp}\frac{100}{1+{(\frac{[S]}{K_{o}})}^{4}}$

Thank you for bringing this to our attention. We added the parameter *K*_0_ as suggested fixing it at *K*_0_ = 1 so that it does not affect our analyses.

3. (Page 17) Equation (63): Should be corrected as (1 + *k*_4_)A = A*_T_* − *K*˜3AR Corrected.

### Minor Comments, Main Text:

1. (Page 4) Equation (E): Representation of *f* (*R*) has mistakes: It should be 2*K*˜1 instead of 2*K*˜2, and *R* should be replaced by *R_T_* .

1

We corrected the representation of *f* (*R*) in Equation (E) by replacing the 2*K*˜2 with 2*K*˜1.

1

However, we kept *R* instead of *R_T_* like in previous work (Kim and Forger, 2012; Forger, 2017)

where they use *R* as a surrogate measure for *R_T_* . We added a note at the end of Section 5 in Supplementary Information saying that we use *R* in place of *R_T_* in the main text.

2. (Page 5) Equation (N): Representation of *f* (*R*) has mistakes:

*K*˜2(1 + *k*_4_).

Corrected.

*K*˜2	should be replaced by

3. (Page 7) Equation (3): Representation of AR has mistakes: *K*˜2 should be replaced by *K*˜2(1 +

*k*_4_).

Corrected.

4. (Page 9) Figure 1D caption should be corrected as “The core model generates oscillations for a ~~higher~~ lower fraction of parameter sets as *K_d_* increases from 10*^−^*^4^ to 10*^−^*^1^.

Corrected.

5. Figure 1D: Better if the y-axis labeling is more specific (“Fraction of parameter sets”).

Fixed. We also made additional changes to the figures to increase consistency across the panels and figures.

### Minor Comments, Supplementary Information:

We thank the reviewer for the very thorough review of the equations and text in the Supplementary Information. These comments helped to significantly improve the reading and clarity of our work.

1. (Page 2) Section 1, Paragraph 1, line 4: ... phosphorylation sites on KaiC-Ser-431 (S431) and KaiC-Thr-432 (T432).

Corrected.

2. (Page 4) Equation (9): [UB] should be [BU] to be consistent with the notation. Corrected.
3. (Page 6) Figure 3E,F: Units of the ratios does not match in both figures. Corrected. This figure has been moved to the main text and is now Figure 2.
4. (Page 7) Supp. Figure 3 caption, line 5: (G-F) should be (G-H). Corrected.
5. (Page 8) Equation (13), Lines 3 and 4: *A_t_* should be replaced by *A_T_* . Corrected.
6. (Page 9) Supp. Figure 5, A: *C_T_* is mentioned as *ν* in the figure. (*C_T_* ≈ *ν*, but *ν* is not defined anywhere, and *C_T_* is used everywhere in the text and in the caption.

We replaced *ν* with *C_T_* in the figure.

7. (Page 10) Equation (18): *A_t_* should be replaced by *A_T_* in the numerator and term *A_T_* should not be in the denominator. (Page 8, Equation (13), line 4).

Corrected. Yes, based on lines 3 and 4 of Equation (13), there should not be an *A_T_* term in the denominator of both line 4 of Equation (13) and in the referenced Equation (18).

8. (Page 10) Equation (23): In the last term, it should be *^k^*^4^

*k*

1

instead of *^k^*^4^ .

3

*k*

Corrected.

9. (Page 10) Equation (24): It should be $(1+ \frac{k_{4}}{k_{2}}+ \frac{k_{4}}{k_{3}}$ ) instead of $( \frac{k_{4}}{k_{2}}+ \frac{k_{4}}{k_{3}})$

Corrected.

10. Page 11) Jacobian Matrix *J*_2_: Close of brackets missing in the first row, second column term. Corrected.
11. (Page 11) Equation (29), Line 2: Typo in the last term. It should be as: *bk*^2^.

2

Corrected.

12. (Page 11) Equation (30), Line 1 and 2: Last term should be negative instead of positive. (Line 1: −3*bk*^2^, Line 2: −3*bk*_2_). From line 1 to 2, *k*_2_ term is factored, therefore line 1 is not equal to line 2 (Suggest using =⇒ ).

2

We added =⇒ to line 2 to make it clear that it is not equal. Also, the negative signs for 3*bk*_2_ were fixed in lines 1 and 2.

13. (Page 12) In TTFL Equations: Equation for [A], term *A_T_* should not be in the denominator. Corrected.
14. (Page 13) In PTR Equations: Equation for [A], term *A_T_* should not be in the denominator. Corrected.
15. (Page 14) Table A, row 2: Unit of *k^f^*

2

should be 1*/µMhr*.

Corrected.

16. (Page 14) Table A, row 8, 9, 10: Unit of $k_{1}^{off},k_{2}^{off}, k_{3}^{off}$should be 1*/hr*.

Corrected.

17. (Page 14) Table B, row 4: Unit of *V_trsp_* for TTFL with new parameter *K*_0_ is *µM/hr*. Unit of *V_trsp_* for PTR is *µM/hr*.

Corrected.

18. (Page 14) Table B, row 8: Unit of *K_d_* is *µM* , not 1*/µM* . Corrected.
19. (Page 15) Equation (38): Following two typos should be corrected: $AR_{p}=\frac{k_{2f}}{k_{2r}+k_{3}}AR$

Corrected.

20. (Page 17) Equation (51): Last term should be positive, not negative. (+*k*_4_*_f_ A*) Corrected.
21. (Page 17) Equation (55) and Equation (56): *AR_p_* should be corrected as *A_p_R_p_*. Corrected.
22. (Page 17) Equation (57): Should be corrected as: : $A_{p}R_{p}=\frac{k_{2f}}{k_{2r}+k_{3}}AR$

Corrected.

23. (Page 17) Equation (58): Should be corrected as: *A_p_* = *^k^*^3^ *A_p_R_p_* + *^k^*4*f A*.

Corrected.

*k*4*r*

*k*4*r*

24. (Page 17) Equation (60): where *K*˜1 is defined as ~~above~~ in (42). Corrected.
25. (Page 18) Equation (65): Should be corrected as: $\tilde{K}_{2}AR=\frac{(A_{T}-\tilde{K}_{3}AR)(R_{T}-\tilde{K}_{1}AR)}{(1+k_{4})}$

Corrected.

26. (Page 18) Equations (66) to (68): In all the equations *K*˜2 should be replaced by *K*˜2(1 + *k*_4_). Corrected.

# Reviewer 2:

The authors analyze conceptual models of circadian rhythms and extract several interesting features that contribute to self-sustained oscillations beyond the classical Goodwin concept with inhibition via unrealistically high Hill coefficients. I find the following aspects very interesting: balance of acti- vator and inhibitor (following Kim/Forger), fine-tuning of the balance via auxiliary transcriptional feedbacks (proposed also in Kim/Forger), an essential role of sequestration (discussed by several authors earlier), and of phosphorylation (leading to inactive proteins that could replace degradation as pointed out by Larrondo). Most of these ideas are not really new. In the manuscript, however, interesting mathematical results are presented combined with extensive simulations with randomly selected parameters. I find also the comparative approach (cyanobacteria, Neurospora, mammals) of interest since it indicates that the above principles might represent generic features.

We thank the reviewer for the summary of our work and for the thorough review that helped us to improve our work. We agree that there are some aspects of the present manuscript that are found independently in other manuscripts. However, we believe that the significance of our work is in the synthesis of previous experimental and computational approaches and in the extension of previous work to compare seemingly common design principles in seemingly divergent organisms.

Despite these positive aspects I cannot support the idea to present the results in a Short Report with extensive supplements. The condensation of all the results to a few pages with just 2 figures makes the paper inaccessible to most readers. In my eyes, the manuscript is only understandable to readers familiar with the clocks in cyanobacteria, fungi, and mammals, with the ideas of sequestration, and with previous studies such as the Kim/Forger paper.

We acknowledge that the short report format may not be appropriate for this work as the journal audience may not have enough mathematical background. Thus, after talking with the editor, we have augmented the manuscript to a research article. We moved some of the content from the Supplementary Information to the main article (e.g., the detailed cyanobacterial clock). We also added exposition and details in the main article to help the reader follow the story more easily.

**Specific comments:**

1. The idea of KaiA sequestration with already described in a modeling papers by Clodong et al. (MSB 2007) and Axmann et al. (Genome Inf. 2007). The replacement of high Hill coefficients has been discussed in a convincing manner already by Gonze (PLoS One 2013). Sequestration as substitute of direct feedbacks has been discussed extensively in papers by S. Legewie and in Jolley et al. (Cell Reports 2012). Inactive complexes in fungi and mammals controlled by phosphorylation have been discussed extensively by Aryal et al. (Mol Cell 2017), Chiou et al. (PNAS 2016), Upadyuy et al. (Sci Reports 2020), Partch (JMB 2020).

We thank the reviewer for bringing these references to our attention, and we apologize for any oversight. We have added references where appropriate.

2. I find the explanation of Eqns. (C) insufficient. Only with the aid of Fig. 1 and of the Kim/Forger paper readers can follow.

We have added a reference to Section 3 in the Supplementary Information where there is a derivation of the repression function in System (C). We are avoiding putting technical details in the main portion of the manuscript to increase readability for those with less mathematical background.

3. ”*r* is a constant of order one” is confusing.

We have revised the text to read: “*r* is a constant” to avoid any confusion for the reader.

4. *C_T_ >* 3*A* might be simpler than introducing epsilon.

We thank the reviewer for this suggestion. To make the section more readable, especially to readers with less mathematical background, we have decided to remove the epsilon from Condition (1). When taking *E <<* 1 as we did, the effect due to epsilon is trivial. The important result of Condition (1) resides in the linear relationship *C_T_ >* 3*A_T_* , as reflected in the figures.

5. Since there are no clear homologies between clock proteins in cyanobacteria, fungi, and mam- mals I suggest to avoid the term evolution. It is more the use of common minimal design principles than a real evolution.

We qualify our statements about evolution with the term convergent, e.g., “. . . molecular clock mechanisms in divergent species may have evolved *convergently.*”. In this way, we are not suggesting that these mechanisms evolved divergently from some first ancestor but rather the mechanisms evolved separately but reflect common design principles as the reviewer rightly states.

6. The left part of Fig. 2C is not informative.

We have updated Figure 4C (see below) to include the percent of parameter sets that gen- erate oscillations for more *K_d_* values. In particular, we plot how varying the strength of dephosphorylation versus phosphorylation affects the generation of oscillations at *K_d_* = 0*.*0001*,* 0*.*001*,* 0*.*01*,* 0*.*1*,* and 1. With this change, the old left side becomes important as, at a smaller *K_d_* value, the system is more likely to generate oscillations with stronger dephos- phorylation.

**Second round of review**

**Reviewer 1**

The revised manuscript addressed most of my concerns with appropriate backgrounds and explanations of the presented data, which improved readability of the manuscript. I only have a few minor comments (below). 1. Figure 2A shows D-KaiC, but D-KaiC is not defined in the manuscript. I assumed that D-KaiC is the unphosphorylated KaiC. 2. Figure 4B: It is interesting to observe non-monotonic changes of sensitivity of f(R) as the abundance of R changes at different values of k2. Please provide rationale for this observed data. 3. Page 8, line 16: Please elaborate on this sentence “Even though the assumptions used to derive the Hill function are very restrictive.” If this refers to usages of high Hill coefficient, then this is incorrect, because high Hill coefficient can be reduced with additional variables in the model. For example, one of the cited references (Hong et al, 2008) used a Hill coefficient of 2, which is biologically feasible

**Authors’ response to reviewers**

Reviewer 1 Comments

1. Figure 2A shows D-KaiC, but D-KaiC is not defined in the manuscript. I assumed that D-KaiC is the unphosphorylated KaiC. We apologize for this error. The D-KaiC was carried over from the original paper presenting the data. The D-KaiC label was changed to ST to match the Figure 2B labels and our notation in the rest of the manuscript.
2. Figure 4B: It is interesting to observe non-monotonic changes of sensitivity of f(R) as the abundance of R changes at different values of k2. Please provide rationale for this observed data.

The sensitivity is a measure of how the transcription rate changes due to a small change in the repressor R. The non-monotonicy of the sensitivity is important because it reveals that the rate of transcription does not change much away from the steady state value of R. However, once you are close to the steady state value, transcription drastically changes, and if it is sensitive enough at that value, sustained oscillations are possible.

3. Page 8, line 16: Please elaborate on this sentence “Even though the assumptions used to derive the Hill function are very restrictive.” If this refers to usages of high Hill coefficient, then this is incorrect, because high Hill coefficient can be reduced with additional variables in the model. For example, one of the cited references (Hong et al, 2008) used a Hill coefficient of 2, which is biologically feasible

This comment does not refer to usages of high Hill coefficients. Rather, we are referring to the assumption in the derivation that binding of the protein occurs only on the promoter and that repressor proteins bind rapidly with the promoter. The Forger 2017 reference we cite lists these assumptions as restrictive, not necessarily that of a high Hill coefficient.
